# Effect of Overweight/Obesity on Traumatic Dental Injuries in Children and Adolescents: A Systematic Review with Meta-Analysis

**DOI:** 10.3390/jcm15197749

**Published:** 2026-10-08

**Authors:** Anika Islam, Spyridon N. Papageorgiou, Blend Hamza, Marc Schätzle

**Affiliations:** Clinic of Orthodontics and Pediatric Dentistry, Center for Dental Medicine, University of Zurich, Plattenstrasse 11, 8032 Zurich, Switzerland

**Keywords:** children, obesity, overweight, systematic review, traumatic dental injury, meta-analysis

## Abstract

**Background/Objectives**: Overweight and obesity are growing global pediatric health concerns, but their association with the occurrence of traumatic dental injuries (TDIs) is unclear. The aim of this systematic review was to critically appraise evidence on the prevalence of TDIs in children and adolescents according to their weight. **Methods**: Systematic literature searches without restrictions were undertaken in six databases from inception up to August 2026 for studies reporting on the association between overweight/obesity and TDIs among children. After duplicate study selection, data extraction, and risk of bias assessment with a custom tool based on the Joanna Briggs Institute’s tool for case-control studies, random-effects meta-analyses were performed, followed by subgroup, meta-regression, and sensitivity analyses. **Results**: Twenty-six studies covering a total of 23,772 patients (54.5% male; average age 10.1 years) were included. Increased patient weight (overweight/obesity) was significantly associated with higher odds of TDIs in children (Odds Ratio [OR] 1.48; 95% CI 1.22 to 1.80; *p* < 0.001). TDIs were significantly associated with obesity (OR 1.45; 95% CI 1.10 to 1.96; *p* = 0.01) but not with overweight (OR 1.13; 95% CI 0.79 to 1.62; *p* = 0.45). No significant effects were seen for age, sex, dentition, confounder adjustment, study design, and study sample size, while differences were seen according to the TDI diagnostic criteria used. The certainty in existing evidence was very low due to methodological limitations of existing studies and between-study heterogeneity. **Conclusions**: Obesity seems to be associated with increased odds of TDIs in children and adolescents, whereas overweight alone shows no significant association. However, due to the very low certainty in the evidence, these results should be interpreted cautiously and warrant further high-quality research.

## 1. Introduction

### 1.1. Background

Traumatic dental injuries (TDIs) are a common oral health problem in children and adolescents and may have important functional, aesthetic, psychological, and social consequences. Their clinical management can range from simple restorative procedures to complex and long-term treatment, particularly when injuries involve the dental pulp or supporting periodontal tissues. The occurrence of TDIs is multifactorial, reflecting interactions between individual characteristics, dental anatomy, behavior, and environmental exposures. Established factors associated with a greater likelihood of TDI include male sex, increased incisal overjet, inadequate lip coverage, anterior open bite, participation in sports, and a previous history of dental trauma [1,2]. Additionally, overweight has also been identified among the factors associated with a greater chance of TDIs, although it highlighted considerable limitations in the methodological quality of the available evidence [1].

Overweight and obesity may be relevant to TDI through several potential pathways. Excess body weight during childhood and adolescence has been associated with poorer motor performance and may affect balance, coordination, and physical mobility, potentially increasing susceptibility to falls and other accidental events [3]. These mechanisms are particularly relevant to dental trauma because falls and collisions are important causes of TDI in young people. In addition, differences in body mass may influence the biomechanical characteristics of an impact and consequently the magnitude of forces transmitted to the craniofacial region. Thus, overweight and obesity could plausibly influence the risk of TDI, either directly through biomechanical mechanisms or indirectly through an increased propensity for falls and accidents. However, these mechanisms should be interpreted as hypotheses supporting an association rather than evidence of a causal relationship.

Epidemiological studies investigating the relationship between excess body weight and the occurrence of TDIs have produced inconsistent findings. The first meta-analysis specifically addressing this question, published in 2015 [4], included 17 observational studies involving children or adolescents and found a statistically significant association between overweight/obesity and TDI (pooled OR 1.30; 95% CI 1.11 to 1.53). Nevertheless, substantial heterogeneity was observed among the studies, indicating that the magnitude of the association may vary according to population characteristics, study design, definitions of overweight/obesity, and methods used to ascertain TDIs. Subsequent evidence has remained heterogeneous, and a 3-year prospective study of 785 adolescents [5] reported that participants classified as overweight/obese had a 2.78-fold greater likelihood of developing TDI after adjustment for socioeconomic status, lip coverage, incisal overjet, and previous trauma. Conversely, a later case-control study of preschool children found that obesity, but not overweight, was associated with TDI (OR 2.05; 95% CI 1.14 to 3.67), further suggesting that the association may differ according to age and the definition of excess body weight [6].

Given the increasing prevalence of overweight and obesity among young people and the potentially preventable consequences of TDI, clarifying the relationship between excess body weight and dental trauma is clinically and epidemiologically relevant. Importantly, the previous meta-analysis [4] was published in 2015, did not formally assess the effect of confounder adjustment across studies, and included evidence published up to 2014, whereas additional observational studies have subsequently been published. Moreover, an updated overview of TDI risk factors identified overweight as a potential risk factor but emphasized the generally low methodological quality of the underlying systematic-review evidence [1]. Therefore, an updated and methodologically rigorous synthesis is warranted to determine whether overweight and/or obesity is associated with an increased risk of TDI in children and adolescents and to investigate potential sources of heterogeneity across studies. The present systematic review and meta-analysis was undertaken to synthesize the available evidence on the association between overweight/obesity and traumatic dental injuries in this population.

### 1.2. Objective

The aim of this systematic review was to critically assess the evidence from observational clinical studies on children and adolescent on the effect of overweight/obesity on TDIs. The null hypothesis was that there is no difference in the prevalence of TDIs between children with increased weight (overweight/obese) and children without increased weight.

## 2. Material and Methods

### 2.1. Protocol Development and Focused Question

**Figure 1:**
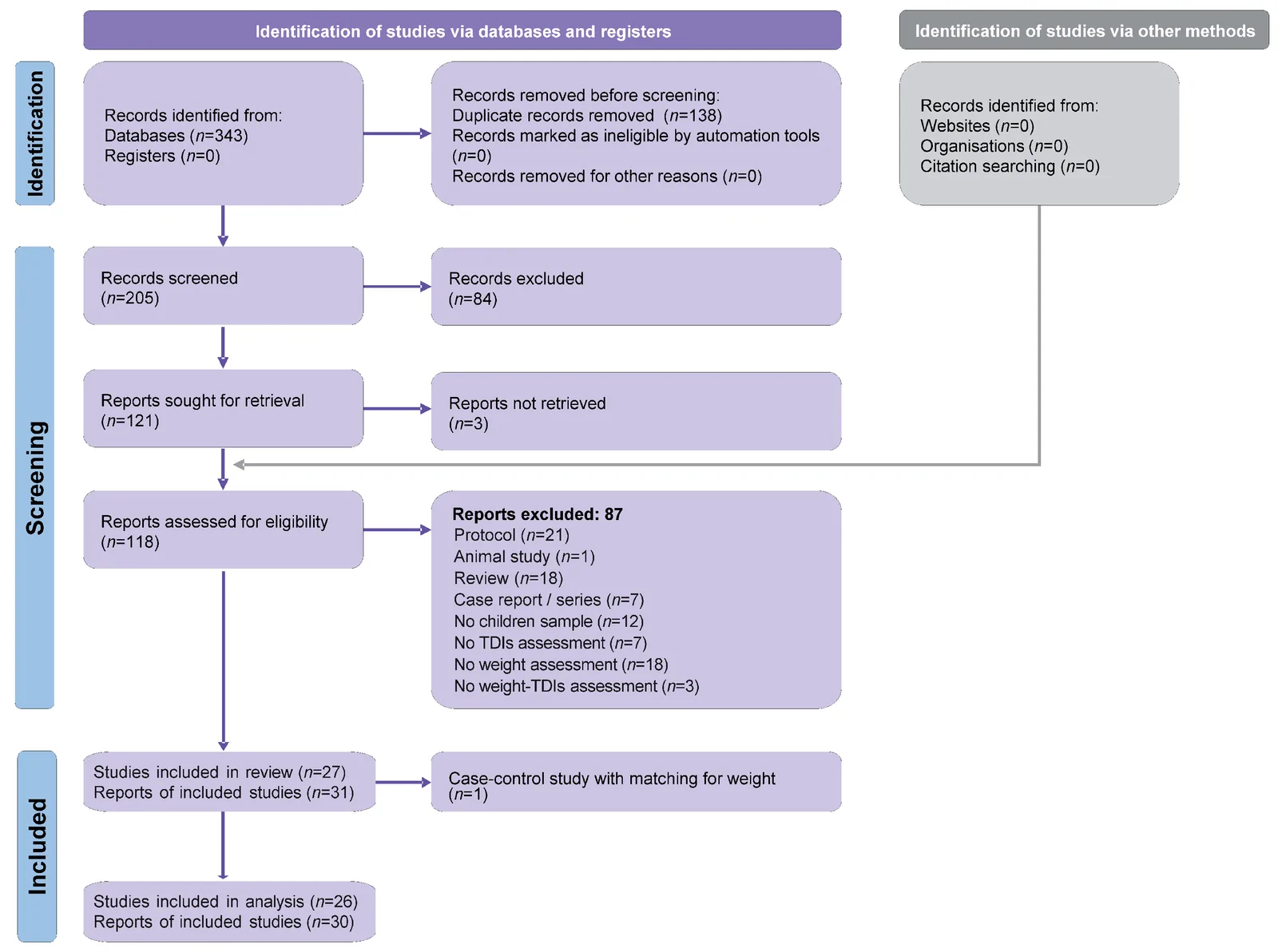
PRISMA flow diagram for the identification and selection of studies included in this systematic review.

This review’s protocol was made a priori, registered in PROSPERO (CRD420261475968), and all post hoc changes were transparently reported (File S1). The conduct and reporting of this review are guided by the Cochrane Handbook [7] and the PRISMA statement [8], respectively (Figure 1).

### 2.2. Eligibility Criteria

Based on the Population–Exposure–Comparison–Outcome Study (PECOS) design schema, experimental and observational (randomized and non-randomized) comparative studies (S) on underage (<18 years of age) patients of any sex, ethnicity, or systemic disease (E) having increased body weight (characterized as overweight or obese) (C) compared to patients without increased body weight (either normoweight or underweight) and assessing TDIs (O) were included, without any limitations in language, publication year, or status (S).

The inclusion of non-randomized comparative studies was judged imperative due to the nature of the research question, since overweight/obesity cannot be randomized to patients. Non-clinical studies, case reports/series (defined as studies with <10 patients), animal studies, and studies that did not assess TDIs in conjunction with patient body weight were excluded. The prevalence of TDIs was the primary and sole outcome, and no further outcomes were assessed.

### 2.3. Information Sources and Search

Six electronic databases were searched without restrictions from inception to August 2026 (Table S1), while open-access databases for gray literature (Directory of Open Access Journals, Digital Dissertations, metaRegister of Controlled Trials, WHO, Google Scholar) and the reference/citation lists of included articles or systematic reviews were manually searched for additional studies.

### 2.4. Study Selection, Data Collection, and Risk of Bias

After de-duplication, two authors (AI/MS) screened the titles/abstracts of retrieved hits to exclude obviously inappropriate studies, before moving to full-text assessment. Any differences between the two reviewers were resolved by discussion with another author (BH).

Data extraction was performed independently by two authors (AI/MS) in the same way to resolve discrepancies using pre-defined/piloted forms covering: (a) study characteristics (design, clinical setting, country); (b) patient characteristics (age, sex, systemic disease); (c) TDI criteria used; (d) weight categories assessed and definitions; and (e) potential confounders taken into account during statistical analysis. Data was extracted from published reports, and no corresponding authors of included studies were contacted to request additional data.

The risk of bias of both randomized trials and observational studies was assessed with a modified version of the JBI tool for case-control studies [9], according to the nature of the research question, regardless of the design of available studies. All studies were appraised independently by two authors (AI/MS), with any differences discussed with a third author (BH).

### 2.5. Data Analysis

As the effect of overweight/obesity on TDIs was bound to be affected by demographic and morphology-related characteristics, a random-effects model was deemed appropriate a priori based on clinical and statistical reasoning [10], to calculate the average distribution of effects and a restricted maximum likelihood variance estimator with the Hartung-Knapp correction [11]. Odds Ratios (ORs) with their 95% Confidence Intervals (CIs) were used to pool data from included published papers. The extent and impact of between-study heterogeneity were assessed by inspecting the forest plots and by calculating the τ^2^ (absolute heterogeneity) or the I^2^ statistics (relative heterogeneity; proportion of total variability explained by heterogeneity and not chance), while also considering the direction of heterogeneity (localization on the forest plot) and uncertainty around heterogeneity estimates [12]. Between-study heterogeneity was incorporated into 95% prediction intervals to estimate possible effects in a future scenario [13].

Possible sources of heterogeneity were sought through random-effects subgroup/meta-regression analyses for meta-analyses with ≥5 studies according to patient sex (% male in the sample of each study), mean age, and dentition of the included patients. Reporting bias assessment was conducted for meta-analyses with ≥10 studies using contour-enhanced funnel plots and formally tested for asymmetry with the Thompson test.

The overall strength of clinical recommendations was rated using the Grades of Recommendations, Assessment, Development and Evaluation (GRADE) approach [14,15]. The forest plots produced were augmented with contours denoting the magnitude of the observed effects to assess heterogeneity, clinical relevance, and imprecision [16]. Four sensitivity analyses were conducted: one according to the sample size of included studies, one according to the design of included studies, one according to the TDI criteria used in each included study, and one according to whether crude estimates or estimates adjusted for confounders were used in each study.

Analyses were performed in R (version 4.4.1; R Core Team, 2024) by one author (SNP) using an open dataset [17]. All *p* values were two-sided with an alpha of 5%, except for between-studies or between-subgroups heterogeneity, where alpha was set at 10% [18].

## 3. Results

### 3.1. Study Selection

A total of 343 hits were retrieved by the electronic database search (Figure 1; Tables S1 and S2). After removing duplicates, 205 full-text papers were checked against the eligibility criteria. In the end, 31 publications pertaining to 27 unique studies were included in this review’s qualitative synthesis [5,6,19,20,21,22,23,24,25,26,27,28,29,30,31,32,33,34,35,36,37,38,39,40,41,42,43,44,45,46,47].

### 3.2. Characteristics of Included Studies

Of the twenty-eight included studies, three were longitudinal cohort studies [5,22,25], two were case-control studies [6,34], and the remaining twenty-three were cross-sectional studies (Table 1).

Included studies were conducted in schools, primary health units, national sports associations, and university clinics in eleven countries (Brazil, Great Britain, India, Italy, Japan, Jordan, Libya, Saudi Arabia, Spain, Syria, and the United States of America). The vast majority of studies (96%; 26/27) did not use systemic health as an eligibility criterion, and only one study [24] specifically included patients with special health care needs. The identified studies included 28,215 patients in total (median sample 764 patients/study; interquartile range 458 to 1134 patients/study). Samples in the included studies were evenly distributed between sexes, with 54.5% being boys (15,368/28,215 from 27 studies reporting sex). The average age of the included patients was 10.1 years (from the 23 studies reporting mean age), while ten studies included patients of a specifically defined age (most often 12 years of age).

The criteria used for the diagnosis/categorization of TDIs were most often those of Andreasen (*n* = 11), those from the UK Children’s Dental Health Survey (*n* = 5), those from the World Health Organization modified by Andreasen (*n* = 3), or others. One included study did not include clinical examination to determine the presence of TDIs, but relied on self-reported diagnoses from the patients or their families [46].

The weight of the included children was assessed in the vast majority of studies (93%; 25/27) through measurement of the Body Mass Index (BMI), while two studies used weight-for-age alone or together with height-for-age [40,41]. Children in included studies were categorized as underweight, normoweight, overweight, or obese, though different rules/cut-offs were used and different categories were often presented pooled together.

Potential confounders in the relationship between increased weight and TDIs were measured and adjusted for in the statistical analysis in 63% (17/27) of the included studies, though great variability in the number and importance of reported confounders existed.

### 3.3. Risk of Bias Within Studies

The assessment of methodological limitations and potential risks for bias of the included studies, according to the JBI tool, are presented in summary in Table 2 and in detail in Table S3.

The most problematic detail was the lack of measuring potential confounders (problematic in 48% of included studies) or adjusting for them in the statistical analyses (problematic in 73% of included studies). Further issues pertained to the short exposure period (as young children under 6 years of age were included; 19% of studies), inclusion of limited sample sizes (19% of studies), lack of a standardized assessment method for TDIs (15% of studies), and lack of a valid assessment method for increased body weight (7% of studies). Overall, six studies (22%) were at low risk of bias, one (4%) was at moderate risk of bias, and the remaining twenty (74%) were at high risk of bias.

### 3.4. Data Synthesis

One study [34] was further excluded from the meta-analysis, since it was a case-control study where the groups with and without TDIs were matched, and therefore the relationship with increased weight would be biased. Therefore, a total of 30 publications pertaining to 26 unique studies were included in the quantitative data synthesis (Table 3).

Three syntheses were performed (Table S4): (a) one analysis assessing the most comprehensive increased weight category (overweight, obese, or their combination); (b) one assessing the association with overweight; and (c) one assessing the association with obesity.

**Figure 2:**
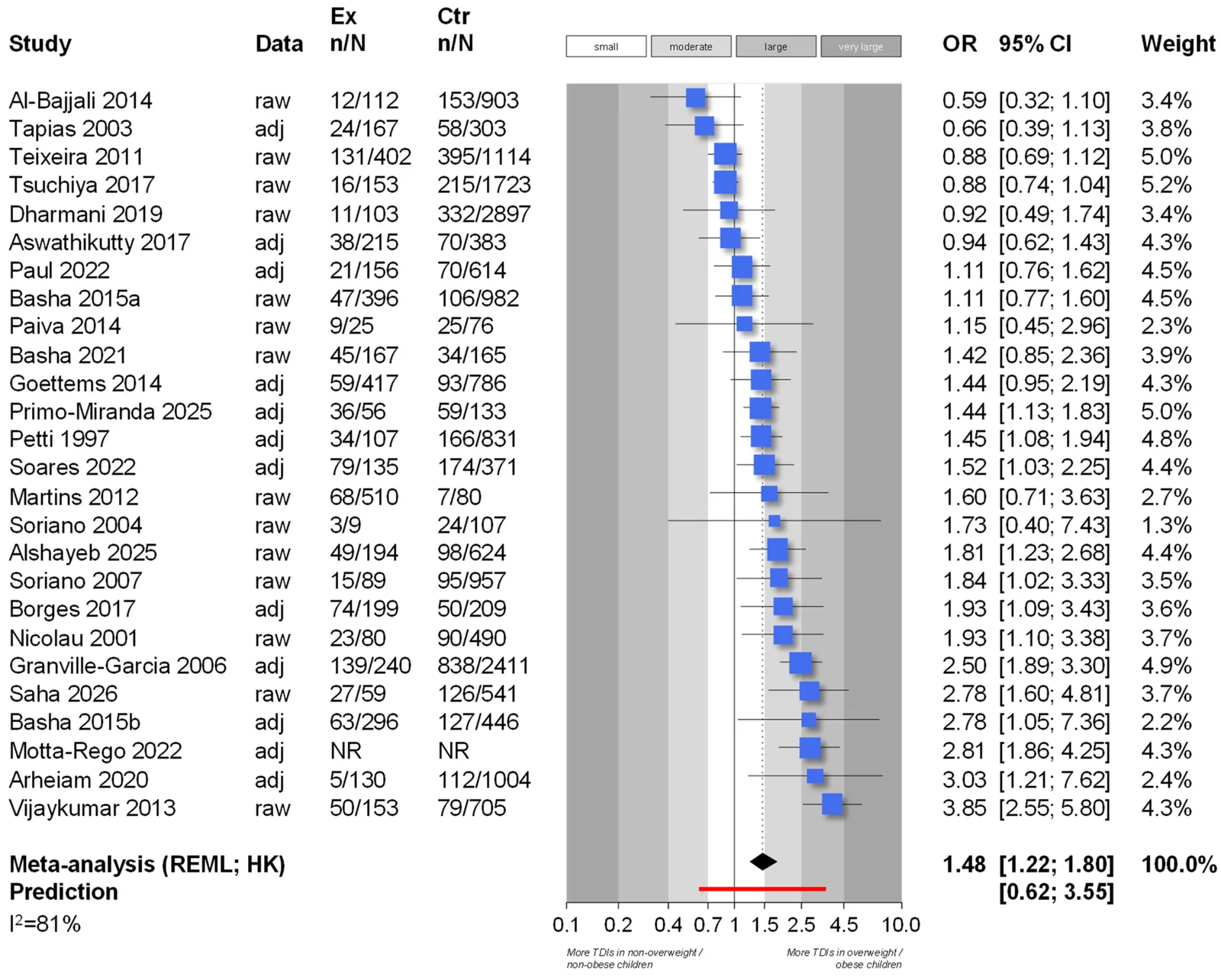
Meta-analysis on the effect of overweight/obesity on the prevalence of traumatic dental injuries in children. CI, confidence interval; Ctr, control category (underweight/normoweight/overweight); Ex, experimental category (overweight/obese); HK, Hartung–Knapp correction; n/N, events/sample; NR, not reported; OR, odds ratio; REML, restricted maximum likelihood; TDI, traumatic dental injury.

**Figure 3:**
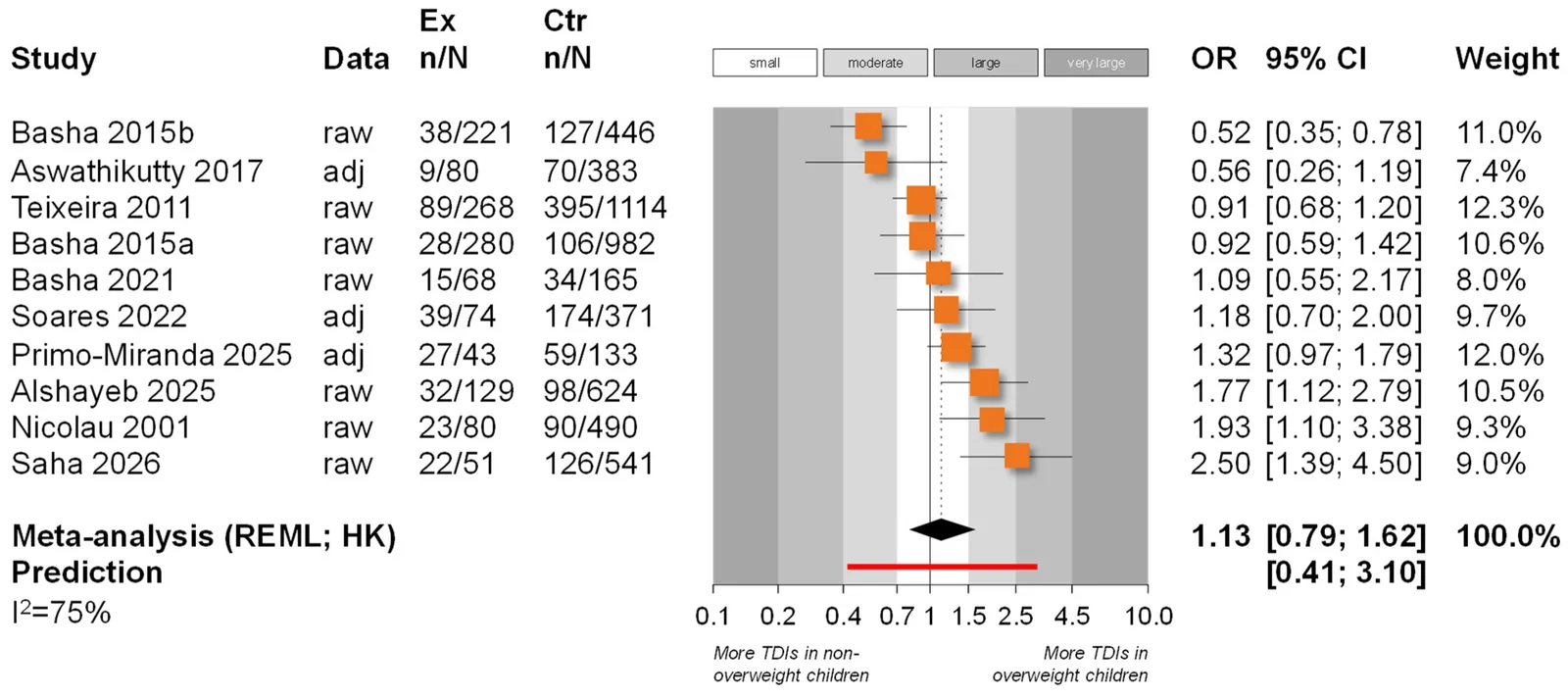
Meta-analysis on the effect of overweight on the prevalence of traumatic dental injuries in children. CI, confidence interval; Ctr, control category (underweight/normoweight); Ex, experimental category (overweight); HK, Hartung-Knapp correction; n/N, events/sample; NR, not reported; OR, odds ratio; REML, restricted maximum likelihood; TDI, traumatic dental injury.

**Figure 4:**
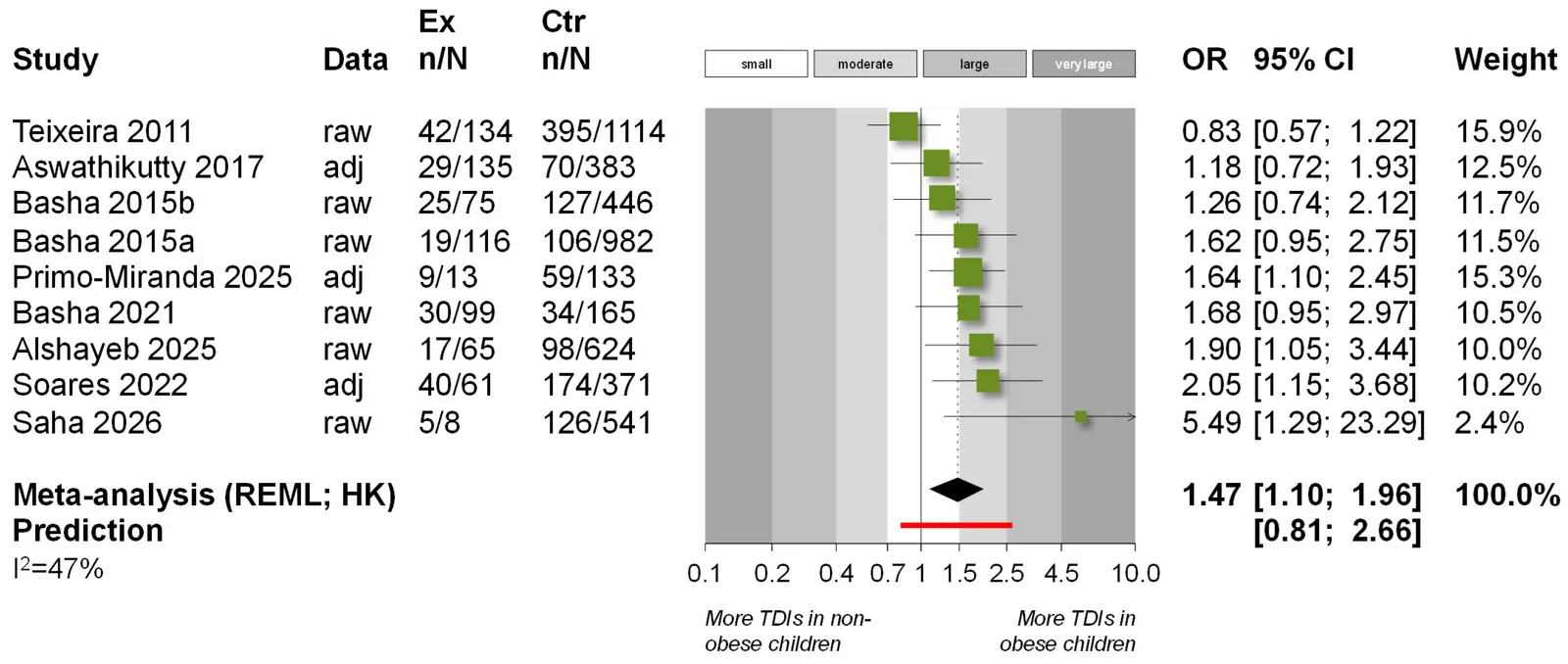
Meta-analysis on the effect of obesity on the prevalence of traumatic dental injuries in children. CI, confidence interval; Ctr, control category (underweight/normoweight); Ex, experimental category (overweight); HK, Hartung-Knapp correction; n/N, events/sample; NR, not reported; OR, odds ratio; REML, restricted maximum likelihood; TDI, traumatic dental injury.

A meta-analysis of 26 studies (23,772 children) indicated that children with increased body weight (overweight or obese) were on average significantly more likely to have experienced TDIs compared to children with lower body weight (underweight or normoweight), with an OR of 1.48 (95% CI 1.22 to 1.80; *p* < 0.001; Figure 2; Table 3). Substantial between-study heterogeneity was observed in relative terms (I^2^ = 81%), and the prediction interval (0.62 to 3.55) indicated that not all overweight/obese patients are associated with higher odds of TDI. This association was not seen for overweight children, where no statistically significant association was seen (10 studies; 6543 children; OR 1.13; 95% CI 0.79 to 1.62; *p* = 0.45; I^2^ = 75%; Figure 3). However, obesity was associated on average with increased odds of TDIs compared to normoweight or underweight patients (9 studies; 5465 children; OR 1.45; 95% CI 1.10 to 1.96; Figure 4). Moderate between-study heterogeneity was seen (I^2^ = 47%), and the prediction interval again included values from both sides of the forest plot—indicating that not all obese children are associated with higher odds of TDI.

Meta-regressions according to patient sex or mean age (Table 4) did not find any significant modifications for any of the meta-analyses performed (*p* > 0.10 in all instances). Subgroup analyses according to dentition (Table S5) did not find any significant differences between patients in the deciduous or permanent dentition.

The assessment of reporting biases (including the possibility of small-study effects and publication bias) could be performed only for two of the three meta-analyses that included at least 10 studies. No obvious funnel plot asymmetry was seen for the association either with overweight/obesity (Figure S1) or overweight (Figure S2), which was also confirmed by the non-significant results of the Thompson test (*p* = 0.41 and *p* = 0.84, respectively).

Sensitivity analysis according to sample size (Table S6) did not indicate systematic differences among small, moderate, and large studies for any of the three meta-analyses (*p* values of 0.29, 0.19, and 0.33, respectively). Sensitivity analysis according to whether a study provided unadjusted (raw) estimates or estimates adjusted for potential confounders (as listed in Table 5) indicated the robustness of all three analyses, since no significant differences were seen (*p* values of 0.59, 0.72, and 0.76, respectively). Sensitivity analyses according to study design (Table S7) did not find differences for the association of overweight/obesity or obesity and TDIs. For the non-significant association of overweight with TDIs, significant differences between study designs were seen (*p* < 0.001), where cohort studies were the only design indicating that overweight was significantly associated with lower odds of TDI (2 studies; 1130 patients; OR 0.53; 95% CI 0.36 to 0.77; *p* = 0.02). Sensitivity analysis according to the diagnostic criteria used for TDIs (Table S8) showed significant differences for all three meta-analyses (*p* < 0.05 in all instances). When looking at studies using the Andreasen criteria, which were the most widely used, the results of all three meta-analyses agreed with the results of the original analysis (indicating significant associations for TDIs with overweight/obesity and obesity, but not overweight alone). For all other criteria used (those from Glendor, the International Association of Dental Traumatology, the United Kingdom Children’s Dental Health Survey, or others), inconsistent results were seen, but these were based on very small subsets of studies (often including only one study).

The overall certainty in the existing evidence was judged to be very low due to the methodological limitations (and the possibility of bias) of the analyzed studies and considerable relative between-study inconsistency (Table 6).

## 4. Discussion

The present systematic review critically appraises evidence from observational clinical studies assessing the association between increased body weight (overweight/obesity) and the occurrence of TDIs among children and adolescents. Evidence from twenty-six studies, including 23,772 children and adolescents, indicated that overweight/obesity was associated with increased odds for dental trauma, and this mostly pertained to obesity, which may be explained by several interrelated biomechanical and behavioral pathways.

Children with overweight/obesity tend to exhibit poorer gross motor coordination, reduced neuromuscular performance, and lower levels of moderate-to-vigorous physical activity, suggesting that excess adiposity may interfere with the motor skills required to maintain stability and respond appropriately to unexpected perturbations [3]. More recent evidence also indicates that greater adiposity is associated with impaired instrumentally assessed postural control in children and adolescents [48], although substantial methodological heterogeneity remains. Such deficits may increase susceptibility to stumbling and falling, which is particularly relevant because falls constitute one of the most common mechanisms of TDI in childhood [49]. Once a fall occurs, greater body mass may also increase the mechanical energy and impact forces involved, while reduced relative muscular capacity and impaired ability to rapidly reposition the limbs may decrease the child’s ability to arrest the fall or protect the face [50]. Alterations in gait, center-of-mass control, plantar loading and sensory feedback associated with excess adiposity may further compromise dynamic balance, particularly during rapid changes in direction or unexpected environmental perturbations [51]. These mechanisms provide a plausible explanation for why obesity, rather than merely being overweight, might confer greater TDI odds, as more severe adiposity may produce greater impairment in motor performance and postural stability. Importantly, the direct epidemiological literature is consistent with this interpretation, where a 3-year prospective study [5] found substantially greater odds of TDI among adolescents with overweight/obesity even after adjustment for socioeconomic status, lip coverage, incisal overjet and previous trauma, while a case-control study in preschool children found that obesity, but not overweight, was significantly associated with TDI (OR 2.05; 95% CI 1.14 to 3.67) [6]. Thus, the association identified in the present study may reflect the combined effects of greater susceptibility to falls, impaired postural and motor control, altered locomotor biomechanics, reduced ability to recover from loss of balance, and potentially greater impact forces when a fall occurs, rather than a single causal mechanism. Nevertheless, these explanations should be interpreted cautiously because the available evidence does not establish causality. Confounding factors such as age, sex, physical activity, socioeconomic status, craniofacial characteristics (particularly overjet and lip coverage), and previous trauma may confound or modify the observed association, even though data from adjusted-for-confounders estimates did not differ from unadjusted ones. Future prospective studies incorporating objective measures of adiposity, postural control, motor competence, physical activity, fall frequency, and characteristics of the traumatic event are therefore needed to clarify the pathways linking obesity to TDIs.

The relationship between overweight/obesity and TDIs may vary according to age and dentition, reflecting differences in motor development, activity patterns, exposure to trauma, and the anatomical characteristics of primary and permanent teeth. TDI is particularly common during periods in which children acquire and refine locomotor skills, while the mechanisms of injury change as children become older and increasingly participate in sports and other activities involving higher-speed movements and collisions. A systematic review of risk factors reported that child age was associated with overall TDI occurrence, although the determinants of trauma differed between primary and permanent dentitions [1]. In primary teeth, falls are the predominant mechanism, accounting for approximately 59% of injuries in a meta-analysis of children aged 0–6 years, whereas increased overjet is the most consistently identified anatomical risk factor [52,53]. In contrast, TDIs in the permanent dentition may be increasingly influenced by sports, recreational activities, and other higher-energy exposures. Age is also clinically relevant because trauma to primary teeth can affect the developing permanent successors; systematic reviews indicate that younger age at the time of primary-tooth trauma is associated with more frequent and severe developmental sequelae in the permanent dentition [54,55]. Therefore, differences in the association between body weight and TDI across age groups or dentitions, although not observed in our analyses, cannot be ruled out and may reflect not only differences in obesity-related motor and biomechanical characteristics but also substantial changes in the underlying epidemiology and mechanisms of dental trauma.

Sex is another important factor when interpreting the association between body weight and TDIs. Male children have generally been reported to experience more TDIs than females, particularly in the permanent dentition, although this difference is less consistent for injuries to primary teeth. An overview of systematic reviews identified male sex as a risk factor for TDI, while a large systematic review and meta-analysis of permanent-tooth trauma reported a pooled OR of 1.52 (95% CI 1.37 to 1.70) for males compared with females [1,56]. Importantly, the sex difference is unlikely to be attributable to biological susceptibility alone and may partly reflect differences in physical activity, participation in contact or high-risk sports, behavior, and exposure to situations in which falls and collisions are more likely. This interpretation is supported by evidence from the primary dentition, where a comprehensive review found that most studies did not identify gender as an independent risk factor after accounting for other variables, whereas increased overjet remained consistently associated with TDI [52]. Accordingly, sex may act as a confounder or effect modifier of the obesity-TDI relationship, particularly if obesity, physical activity, and injury exposure differ systematically between boys and girls—even though no significant association with sex was found in the present review. Future studies should therefore examine sex-stratified associations and investigate whether the relationship between adiposity and TDI remains after accounting for differences in sports participation, physical activity, dentition, and other established risk factors.

The potential relevance of the association between obesity and TDI extends beyond the occurrence of the injury itself because TDI can impose substantial biological, psychosocial, and economic burdens on children and their families. A systematic review and meta-analysis demonstrated that TDI significantly negatively affects oral health-related quality of life (OHRQoL) in both preschool children and schoolchildren, with particularly pronounced effects in the social domain and greater impact associated with more complicated injuries [57]. Similarly, a systematic review focusing on permanent teeth concluded that TDI has a substantial effect on children’s and adolescents’ OHRQoL and that timely treatment can reduce subsequent biological and psychosocial consequences [58]. The economic burden can also be considerable because treatment may require multiple visits, emergency care, restorative procedures, endodontic treatment, follow-up, and, in some cases, long-term rehabilitation. In a prospective one-year cost study of children, the mean total treatment cost was approximately €1351 for non-complex and €1688 for complex injuries, with costs remaining relevant during subsequent follow-up [59]. Earlier prospective data from Sweden similarly demonstrated both direct and indirect costs associated with TDI, including healthcare expenditure, transportation, loss of leisure time, and productivity losses for parents or caregivers [60]. Furthermore, treatment itself appears to improve OHRQoL in children and adolescents affected by TDIs [61]. Consequently, if obesity identifies children at increased risk of TDI, recognizing this association may have preventive implications, although obesity should be considered as one component of a multifactorial risk profile rather than as a stand-alone indication for intervention.

This review also has several limitations. For one, there were various methodological issues in the included studies, which might have introduced bias. Additionally, various definitions were used among included studies for defining overweight/obesity and TDIs, included samples pertained to children of varying age or dentitions, and several different populations were covered. This introduces clinical and statistical heterogeneity. Furthermore, incomplete reporting of results and potential confounders, as well as the lack of open dataset availability [62], has limited the extent of conducted analyses.

## 5. Conclusions

Observational data indicate that obesity, but not overweight, may be associated with higher odds of traumatic dental injuries in children and adolescents. However, the certainty of this evidence is very low across all outcomes due to serious methodological limitations of the included primary studies, unexplained heterogeneity, and the potential for unmeasured confounding. Consequently, the clinical significance and true nature of this potential association remain unclear, underscoring the need for well-designed prospective studies using recommended diagnostic criteria.

## Figures and Tables

**Table 1:** Characteristics of included studies.

Study	Design; Country *; Setting	Systemic Disease	Sample ^†^ (Male%)	Age (Mean; Range)	TDI Definition	Weight Categories	Weight Definition	Confounder Adjustment ^¥^
Al-Bajjali 2014 [19]	CS; JO; School	NR	1015 (46.3%)	12.0; 12.0	WHO modified by Andreasen	Non-obese, obese	BMI	-
Alshayeb 2025 [20]	CS; SY; School	NR	869 (50.4%)	10.5; 9.0–12.0	WHO modified by Andreasen	Under-, normo-, overweight, obese	BMI	-
Arheiam 2020 [21]	CS; LY; School	NR	1134 (48.7%)	12.0; 12.0	WHO modified by Andreasen	Non-overweight, overweight	BMI	Sex, school type, overjet, lip competence, parent education, dental experience
Aswathikutty 2017 [22]	COH; GB; School	NR	598 (46.5%)	15.7: 15.0–16.0	Glendor	Normoweight, overweight, obese	BMI	Sex, age, ethnicity, parental employment, incisor overjet, physical activity, obesity
Basha 2015 [23]	CS; IN; School	NR	1550 (51.5%)	9.5; 6.0/13.0	Andreasen	Under-, normo-, overweight, obese	BMI	Sex, age, socioeconomic status, overjet, lip coverage, school type
Basha 2015 [5]	COH; IN; School	NR	764 (54.1%)	16.0; 16.0	Andreasen	Under-, normo-, overweight, obese	BMI	Socioeconomic status, overjet, lip coverage, trauma history
Basha 2021 [24]	CS; SA; School	SHN	350 (37.4%)	12.0; 6.0–16.0	Andreasen	Under-, normo-, overweight, obese	BMI	Sex, age, overjet, lip coverage, disability type
Borges 2017 [25]	COH; BR; PHU	NR	458 (50.9%)	3.0; 3.0	Andreasen	Normoweight, overweight/obese	BMI	Sex, mother’s schooling, family income, birthweight, birth-length, head circumference, birth type, height
Dharmani 2019 [26]	CS; IN; School	NR	3000 (50.0%)	9.5; 8.0–11.0	Ellis	Non-obese, obese	BMI	-
Goettems 2014 [27]	CS; BR; School	NR	1210 (47.4%)	9.9; 8.0–12.0	UK Children’s Dental Health Survey	Normoweight, overweight/obese	BMI	Sex ^£^, age, family income, maternal schooling, school type, overjet, lip coverage, family structure, household crowding, physical activity, school retention, trauma history
Granville-Garcia 2006 [28]	CS; BR; School	NR	2651 (52.4%)	3.5; 1.0–5.0	UK Children’s Dental Health Survey	Normoweight, overweight/obese	BMI	Sex, age, school type
Martins 2012; 2014 [29,30]	CS; BR; School	NR	590 (46.6%)	NR; 7.0–14.0	UK Children’s Dental Health Survey	Normoweight, overweight/obese	BMI	Sex, age, lip coverage
Motta-Rego 2022 [31]	CS; BR; School	NR	347 (54.8%)	4.1; 3.0–5.0	Andreasen	Under-/normoweight, overweight/obese	BMI	Overjet
Nicolau 2001; 2003 [32,33]	CS; BR; School	NR	764 (49.7%)	13.0; 13.0	UK Children’s Dental Health Survey	Normoweight, overweight	BMI	Sex, family structure, family income, maternal schooling
Nyquist 2018 [34]	CC; US; Uni	NR	58 (43.1%)	10.1; 7.0–14.0	NR	Normoweight, overweight, obese	BMI	-
Paiva 2014 [35]	CS; BR; School	NR	101 (46.5%)	12.0; 12.0	Andreasen	Under-/normoweight, overweight/obese	BMI	-
Paul 2022 [36]	CS; BR; School	NR	770 (48.6%)	14.0; 12.0–16.0	Andreasen	Normoweight, overweight	BMI	Sex, age, school type, nutritional status, dental experience
Petti 1997 [37]	CS; IT; School	NR	938 (48.7%)	NR; 6.0–11.0	Garcia-Godoy	Non-obese, obese	BMI	Sex, age, trauma predisposition
Primo-Miranda 2025 [38]	CS; BR; School	NR	189 (48.1%)	NR; 3.0–6.0	Andreasen	Normoweight, overweight, obese	BMI	Pacifier use, lip coverage, overjet, arch shape, motor delay, mental state
Saha 2026 [39]	CS; IN; School/Uni	NR	600 (53.2%)	NR; 6.0–13.0	IADT	Normoweight, overweight, obese	BMI	-
Soares 2022 [6]	CC; BR; School	NR	506 (50.0%)	4.3; 3.0–5.0	Andreasen	Normoweight, overweight, obese	BMI	Overjet, anterior open bite
Soriano 2004 [40]	CS; BR; School	NR	116 (51.7%)	12.0; 12.0	Andreasen	Non-obese, obese	Weight for age	-
Soriano 2007; 2009 [41,42]	CS; BR; School	NR	1046 (50.3%)	12.0; 12.0	Andreasen	Non-obese, obese	Weight/height for age	Sex ^£^, school type ^£^
Tapias 2003 [43]	CS; ES; School	NR	470 (52.0%)	10.0; 10.0	Other	Normoweight, overweight	BMI	Sex, socioeconomic status, overjet
Teixeira 2011; Dame-Teixeira 2013 [44,45]	CS; BR; School	NR	1528 (50.4%)	12.0; 12.0	UK Children’s Dental Health Survey	Under-, normo-, overweight, obese	BMI	-
Tsuchiya 2017 [46]	CS; JP; NSA	NR	5735 (72.0%)	11.0; 6.0–15.0	Self-reported	Under-, normo-, overweight	BMI	Sex ^£^, school grade, sport type, athletic details, screen/sleep time, trauma history
Vijaykumar 2013 [47]	CS; IN; School	NR	858 (52.7%)	10.8; 10.0–12.0	NR	Normoweight, overweight	BMI	Sex ^£^

CC, case-control study; COH, longitudinal cohort study; CS, cross-sectional study; BMI, body mass index; IADT, International Association of Dental Traumatology; NR, not reported; NSA, national sports association; PHU, primary health unit; SHN, special healthcare needs; Uni, university clinic; WHO, World Health Organization. * Based on the ISO 2-letter code for longitudinal studies, this pertains to the final sample size. ^†^ for longitudinal studies, this pertains to the final sample size. ^¥^ Pertaining specifically to the effect of overweight/obesity on TDIs. ^£^ Used for stratification.

**Table 2:** Risk of bias summary of included studies using the JBI risk of bias tool.

Domain	Yes % (*n*)	Partly % (*n*)	No % (*n*)
Were the same criteria used for identification of normoweight/overweight/obese patients?	93% (25)	0% (0)	7% (2)
Was overweight/obesity measured in a standard, valid and reliable way?	93% (25)	0% (0)	7% (2)
Was overweight/obesity measured in the same way for patients with/without TDI?	100% (27)	0% (0)	0% (0)
Were confounding factors identified?	52% (14)	22% (6)	26% (7)
Were TDIs assessed in a standard, valid and reliable way?	85% (23)	0% (0)	15% (4)
Was the exposure period of interest long enough?	81% (22)	0% (0)	19% (5)
Was the sample size adequate (>350 patients)?	81% (22)	0% (0)	19% (5)
Was appropriate statistical analysis used, including confounder adjustment?	27% (7)	46% (12)	27% (7)

TDI, traumatic dental injury.

**Table 3:** Details of meta-analyses performed.

Comparison	Studies (Patients)	OR (95% CI)	*p*	tau^2^ (95% CI)	I^2^ (95% CI)	Prediction
Overweight/obese	26 (23,772)	1.48 (1.22, 1.80)	<0.001	0.17 (0.08, 0.37)	81% (73%, 87%)	0.62, 3.55
Overweight	10 (6543)	1.13 (0.79, 1.62)	0.45	0.17 (0.05, 0.78)	75% (53%, 87%)	0.41, 3.10
Obese	9 (5465)	1.45 (1.10, 1.96)	0.01	0.05 (0, 0.67)	47% (0%, 75%)	0.81, 2.66

CI, confidence interval; OR, odds ratio.

**Table 4:** Meta-regression of the association with patient sex and age for all meta-analyses.

	% Male (Per 10%)			Mean Age (Per Year)		
Comparison	Studies (Patients)	Coefficient (95% CI)	*p*	Studies (Patients)	Coefficient (95% CI)	*p*
Overweight/obese	26 (23,772)	−0.07 (−0.40, 0.26)	0.67	23 (22,393)	−0.04 (−0.10, 0.01)	0.13
Overweight	10 (6543)	0.02 (−0.91, 0.95)	0.96	8 (5775)	−0.06 (−0.17, 0.05)	0.22
Obese	9 (5465)	−0.04 (−0.75, 0.67)	0.90	7 (4770)	−0.05 (−0.13, 0.03)	0.18

CI, confidence interval.

**Table 5:** Sensitivity analyses according to whether crude or adjusted estimates were used.

	Crude		Adjusted		
Comparison	Studies (Patients)	OR (95% CI)	Studies (Patients)	OR (95% CI)	P_subsets_
Overweight/obese	14	1.41 (1.05, 1.90)	12	1.56 (1.18, 2.08)	0.59
Overweight	7	1.19 (0.72, 1.97)	3	1.07 (0.38, 2.95)	0.72
Obese	6	1.45 (0.89, 2.36)	3	1.56 (0.83, 2.90)	0.76

CI, confidence interval; OR, odds ratio.

**Table 6:** Summary of findings according to the GRADE approach.

	Anticipated Absolute Effects (95% CI)				
Outcome Studies (Patients)	Control ^a^	Experimental	Difference in Experimental	Quality of the Evidence (GRADE) ^b^	What Happens
Association between overweight/obesity and TDIs 26 studies (23,772 patients)	191/1000	258/1000	68 patients more (33 to 107 more)	⊕⊖⊖⊖ very low ^c,d^ due to bias & inconsistency	Overweight/obese children might experience more TDIs
Association between overweight and TDIs 10 studies (6543 patients)	245/1000	269/1000	23 patients more (41 less to 100 more)	⊕⊖⊖⊖ very low ^c,d^ due to bias & inconsistency	No effect of overweight on TDIs
Association between obesity and TDIs 9 studies (5465 patients)	253/1000	329/1000	76 patients more (18 to 146 more)	⊕⊖⊖⊖ very low ^c,d^ due to bias & inconsistency	Obese children might experience more TDIs

Exposure: overweight or obesity; Population: children and adolescents; Setting: schools, primary health units, national sports associations, and university clinics (Brazil, Great Britain, India, Italy, Japan, Jordan, Libya, Saudi Arabia, Spain, Syria, and the United States of America). ^a^ Response in the control group is based on a random-effects meta-analysis of the control proportion. ^b^ Starts from “low” due to the inclusion of observational studies. ^c^ Downgraded by two levels for bias due to the inclusion of observational studies with a serious risk of bias. ^d^ Downgraded by one level for inconsistency, since studies were found on both sides of the forest plot. CI, confidence interval; GRADE, Grading of Recommendations Assessment, Development and Evaluation; TDI, traumatic dental injury.

## Data Availability

Data for this study is available through Zenodo (doi: 10.5281/zenodo.21966692).

## Supplementary Materials

The following supporting information can be downloaded at: https://www.mdpi.com/article/10.3390/jcm15197749/s1, Figure S1. Contour-enhanced funnel plot for the effect of overweight/obesity on traumatic dental injuries in children; Figure S2. Contour-enhanced funnel plot for the effect of overweight on traumatic dental injuries in children. Table S1. Literature searches performed for this systematic review (last search date: 11 August 2026); Table S2. List of studies identified from the literature search with their inclusion/exclusion status; Table S3. Detailed risk of bias assessment of included studies using the JBI tool; Table S4. Details of included studies in each meta-analysis; Table S5. Subgroup analysis according to the dentitions of included studies; Table S6. Sensitivity analysis according to the sample size of included studies; Table S7. Sensitivity analysis according to the design of included studies; Table S8. Sensitivity analysis according to the traumatic dental injury criteria of included studies. File S1: Additional details of the systematic review.
