# Supplementary material for: Effect of Overweight/Obesity on Traumatic Dental Injuries in Children and Adolescents: A Systematic Review with Meta-Analysis
Source: J Clin Med. 2026 Oct 8;15(19):7749. doi: 10.3390/jcm15197749 (PMC13665793; doi:10.3390/jcm15197749)

SUPPLEMENTARY MATERIAL

Effect of overweight / obesity on traumatic dental injuries in children and adolescents: a systematic review with meta-analysis

File S1. Additional details of the systematic review.

- The minimum adequate sample size for assessed studies was adopted as 350 children, according to the performed sample size calculation of an included study.
- For studies reporting separate adjusted-for-confounders estimates for overweight and obese children, these were pooled (fixed-effect) before being fed to the meta-analysis for the effect of overweight / obesity.

Deviations from registered protocol

- A post hoc sensitivity analysis was performed according to the sample size of each included study using arbitrary cut-offs: 0-500 patients, 500-1000 patients, and >1000 patients.

Table S1. Literature searches performed for this systematic review (last search date 11 August, 2026).

Database	Search strategy	Limits	Hits
MEDLINE (via Pubmed)	("traumatic dental injuries" OR "dental trauma" OR "dental injury" OR "dental injuries") AND (overweight OR obes* OR "BMI" OR "body mass index" OR weight)	Include: humans	71
Embase	Same as Pubmed	Include: humans<br>Exclude: Adult patients	103
Cochrane Database of Systematic Reviews	Same as Pubmed		1
Cochrane Central Register of Controlled Trials	Same as Pubmed		23
Web of Science	Same as Pubmed	Dentistry Oral Surgery Medicine	86
Scopus	Same as Pubmed	Dentistry	53
LILACS	Same as Pubmed		6
**TOTAL (with overlap)**			**343**
**TOTAL (without overlap)**			**205**

Table S2. List of studies identified from the literature search with their inclusion / exclusion status.

Nr	Paper	Status
1	[No authors] American Academy of Pediatric Dentistry (AAPD) Reference Manual. Pediatric Dentistry. 2013;35(6):2-407.	Excluded by title
2	[No authors] American Academy of Pediatric Dentistry. Reference Manual 2010. Pediatric Dentistry. 2010;32(6):2-334.	Excluded by title
3	[No authors] Comparative Evaluation of Calcium Silicate-doped Treated Dentin Matrix and Mineral Trioxide Aggregate as Miniature Pulpotomy Biomaterials in Deep Carious Lesions: a Parallel, Double-blind, Randomised Clinical Trial. 2025.	Excluded by title
4	[No authors] Comparing the Effectiveness Between Air-Q Intubating Laryngeal Airway and Ambu® AuraGain™ Laryngeal Mask for Controlled Ventilation in Paediatric Patients: A Randomized Controlled Trial. 2017.	Excluded by title
5	[No authors] Efficacy of Hook Technique Versus Traditional Methods for Single Hand Ventilation in Obese Patients A Randomized Controlled Trial. 2025.	Excluded by title
6	[No authors] Evaluation of the Clinical Performance of Laryseal Clear Versus Ambu Aura 40 Laryngeal Mask Airway for Preschool Children. 2026.	Excluded by title
7	[No authors] Overview. Pediatric Dentistry. 2014;36(6):2-412.	Excluded by title
8	[No authors] SPEAK 2024 International Dental Conference. Journal of Clinical and Diagnostic Research. 2024;18.	Excluded by title
9	Andresen M, Mackie I, Worthington H. The periotest in traumatology. Part I. Does it have the properties necessary for use as a clinical device and can the measurements be interpreted? Dental Traumatology. 2003;19(4):214-7.	Excluded by title
10	April MD, Arana A, Pallin DJ, Schauer SG, Fantegrossi A, Fernandez J, et al. Emergency Department Intubation Success With Succinylcholine Versus Rocuronium: A National Emergency Airway Registry Study. Ann Emerg Med. 2018;72(6):645-53.	Excluded by title
11	Armasu I, Crumpei I, Vasiliu I, Rusu C, Braha E, Zetu I, et al. SHORT syndrome and rhGH treatment - Is it useful? Hormone Research in Paediatrics. 2016;86:479.	Excluded by title
12	Berthold C, Holst S, Schmitt J, Goellner M, Petschelt A. An evaluation of the Periotest method as a tool for monitoring tooth mobility in dental traumatology. Dent Traumatol. 2010;26(2):120-8.	Excluded by title
13	Bhadule SN, Kalaskar RR, Doiphode A. Stress Distribution in Proclined Anterior Teeth of Pediatric Patients With and Without Mouthguard Use: A 3D Finite Element Analysis. Dent Traumatol. 2025;41(2):203-12.	Excluded by title
14	Bosworth OM, Padilla-Azain MC, Adgent MA, Spieker AJ, Wiese AD, Pham A, et al. Prescription Opioid Exposure During Pregnancy and Risk of Spontaneous Preterm Delivery. JAMA Network Open. 2024;7(2):E2355990.	Excluded by title
15	Brännemo I, Dahllöf G, Cunha Soares F, Tsilingaridis G. Impact of an extended postnatal home visiting programme on oral health among children in a disadvantaged area of Stockholm, Sweden. Acta Paediatrica, International Journal of Paediatrics. 2021;110(1):230-6.	Excluded by title
16	Çatak T, Üstün M, Demiray MD, Yıldız FSU. Would you perform this procedure under general anesthesia? A scenario-based comparison of dentist and anesthesiologist approaches. BMC Anesthesiology. 2026;26(1).	Excluded by title
17	Cieślińska K, Karbowska K, Zaborowicz K, Biedziak B. Digital Technologies in Diagnosing Solitary Median Maxillary Central Incisor Syndrome. Children. 2026;13(1).	Excluded by title
18	Couper RT, Monkhouse W, Busutil M, Thompson P. Stroller safety. Med J Aust. 1994;160(6):335-8.	Excluded by title
19	Dash S, Balasubramanian S. Analysis of clinical indicators of quality in patients with endotracheal intubation. Journal of Clinical and Diagnostic Research. 2017;11(6):UC04-UC7.	Excluded by title
20	Dholam KP, Chouksey GC. Squamous cell carcinoma of the oral cavity and oropharynx in patients aged 18-45 years: A case-control study to evaluate the risk factors with emphasis on stress, diet, oral hygiene, and family history. Indian J Cancer. 2016;53(2):244-51.	Excluded by title
21	Díaz S, Abanto J, Boneckër M, Lara JS, Paiva SM, Olegário IC. Impact of clinical stage and location of dental caries lesions on oral health-related quality of life. Braz Oral Res. 2025;39:e125.	Excluded by title
22	Ferreira RC, Pinto RDS, Reis CD, Moura RNV, Aguiar SO, Drummond AMA, et al. Calibration of SB Brasil 2023 examiners: use of technologies associated with the In-Lux method. Braz Oral Res. 2025;39(suppl 1):e045.	Excluded by title
23	Gardner EC. Head, Face, and Eye Injuries in Collegiate Women's Field Hockey. Am J Sports Med. 2015;43(8):2027-34.	Excluded by title
24	Gerdin PO. Clinico-macroscopically observable disturbances of the enamel mineralization of the permanent teeth. II. Account of etiologic factors, including heredity, in the types occurring in a non-selected material of 1873 children aged 8-15 years from a district with drinking water poor in fluorine. Odontologisk tidskrift. 1969;77(5):337-402.	Excluded by title
25	Giraudon A, Bordes-Demolis M, Blondeau B, Sibai de Panthou N, Ferrand N, Bello M, et al. Comparison of the McGrath® MAC video laryngoscope with direct Macintosh laryngoscopy for novice laryngoscopists in children without difficult intubation: A randomised controlled trial. Anaesthesia Critical Care and Pain Medicine. 2017;36(5):261-5.	Excluded by title
26	Goettems ML, Schuch HS, Hallal PC, Torriani DD, Demarco FF. Nutritional status and physical activity level as risk factor for traumatic dental injuries occurrence: a systematic review. Dent Traumatol. 2014;30(4):251-8.	Excluded by title
27	Greenberg JR, Sinclair S, Janssen CA, Krick K, O'Brien M, Flanagan K, et al. An Electronic Screening System for Oral Health Examination and Collection of Critical Data in a Nonclinical Setting: Validation Trial. Compend Contin Educ Dent. 2018;39(5):318-24.	Excluded by title
28	Gupta K, Sabry HA, Dogan A, Coppa ND, McMenomey S, Delashaw JB, et al. Classification of middle fossa floor dehiscence syndromes. Journal of Neurosurgery. 2015;122(3):557-63.	Excluded by title
29	Hansel J, Rogers AM, Lewis SR, Cook TM, Smith AF. Videolaryngoscopy versus direct laryngoscopy for adults undergoing tracheal intubation. Cochrane Database of Systematic Reviews. 2022(4).	Excluded by title
30	Hasanuddin H, Mude AH, Muhammad Ikbal EM, Ruslin M. Navigating challenges in dentomaxillofacial science by integrating genetics, clinical innovation, and educational intervention: Editorial review. Journal of Dentomaxillofacial Science. 2025;10(2):86-90.	Excluded by title
31	Huang Y, Du L, Liu D, Zhang W, Li T, Zheng J. Hyperangulated videolaryngoscope for difficult airway management: A protocol for a systematic review with meta-analysis and trial sequential analysis. BMJ Open. 2025;15(6).	Excluded by title
32	Huo F, Liang H, Gao L, Liang J, Liu H, Feng Y, et al. Laryngeal mask versus intubation on adverse reactions in pediatric retinoblastoma patients. Translational Pediatrics. 2025;14(9):2110-8.	Excluded by title
33	Javaherforooshzadeh F, Gharacheh L. The comparison of direct laryngoscopy and video laryngoscopy in pediatric airways management for congenital heart surgery: A randomized clinical trial. Anesthesiology and Pain Medicine. 2020;10(3):1-6.	Excluded by title
34	Kadam AB, Jamale PB. Clinical trial for comparison of clinical performance of I-Gel with LMA-proseal in elective surgeries. International Journal of Research in Pharmaceutical Sciences. 2020;11(4):5350-6.	Excluded by title
35	Kang J, Lee H, Kim S, Kim HJ, Lee H, Kwon R, et al. Comorbid health conditions in people with attention-deficit/hyperactivity disorders: An umbrella review of systematic reviews and meta-analyses. Asian J Psychiatr. 2024;99:104135.	Excluded by title
36	Karthikeyan P, Vijayan N, Sivanand N, Manivasakan S, Livingstone D. A Novel Custom Fabricated Teeth Guard for Boyle Davis Mouth Gag- A Feasibility Study. Journal of Clinical and Diagnostic Research. 2022;16(1):MF01-MF3.	Excluded by title
37	Kataoka SH, Setzer FC, Gondim E, Jr., Caldeira CL. Impact absorption and force dissipation of protective mouth guards with or without titanium reinforcement. J Am Dent Assoc. 2014;145(9):956-9.	Excluded by title
38	Keresztes D, Mérei Á, Rozanovic M, Nagy E, Kovács-Ábrahám Z, Oláh J, et al. Comparison of VividTrac, King Vision and Macintosh laryngoscopes in normal and difficult airways during simulated cardiopulmonary resuscitation among novices. PLoS One. 2021;16(11):e0260140.	Excluded by title
39	Kilic Ö, Van Os V, Kemler E, Barendrecht M, Gouttebarge V. The 'Sequence of Prevention' for musculoskeletal injuries among recreational basketballers: a systematic review of the scientific literature. Phys Sportsmed. 2018;46(2):197-212.	Excluded by title
40	Kojima T, Yamauchi Y, Watanabe F, Ichiyanagi S, Kobayashi Y, Kaiho Y, et al. Adverse Events Associated with Airway Management in Pediatric Anesthesia: A Prospective, Multicenter, Observational Japan Pediatric Difficult Airway in Anesthesia (J-PEDIA) Study. Anesthesiology. 2025;143(4):835-50.	Excluded by title
41	Kolod EB, Chern I. Primary care of individuals with intellectual and developmental disabilities-an opportunity for education and advocacy. Journal of General Internal Medicine. 2019;34(2):S612.	Excluded by title
42	Lim QY, Taylor K, Dudding T. The effects of modifiable maternal pregnancy exposures on offspring molar-incisor hypomineralisation: A negative control study. Community Dent Health. 2022;39(4):231-9.	Excluded by title
43	Madalena IR, Küchler EC, Reis CLB, Matsumoto MAN, Stuani MBS, Vilalba Paniagua Machado do Nascimento T, et al. Association of PTH and vitamin D-related genes with dental development in Brazilian children: a cross-sectional study. Braz Oral Res. 2025;39:e033.	Excluded by title
44	McCarthy S, Gulabivala K, St George G, Harvey S, Ng YL. Endodontic sequelae associated with repetitive impacts to the dentofacial region during boxing activities. Int Endod J. 2024;57(10):1380-94.	Excluded by title
45	Mégarbané A, Hana S, Mégarbané H, Castro C, Baulande S, Criqui A, et al. Clinical and Molecular Update on the Fourth Reported Family with Hamamy Syndrome. Molecular Syndromology. 2021;12(6):342-50.	Excluded by title
46	Mendes JC, Ceccato MDGB, Reis AMM, Costa AMGD, Pantuzza LLN, Furtado dos Santos S, et al. Active Pharmacovigilance Project on the safety profile of Dolutegravir in Brazil. AIDS Care - Psychological and Socio-Medical Aspects of AIDS/HIV. 2023;35(5):729-38.	Excluded by title
47	Mollabashi V, Soleymani M, Arabestani MR, Farhadian M, Abbasalipourkabir R, Salehzadeh M. Evaluation of Nano TiO2 Modified Orthodontic Composite Effects on S. mutans Population and Enamel Demineralization in Fixed Orthodontic Patients; a Split Mouth Randomized Controlled Clinical Trial. Biological Trace Element Research. 2023;201(10):4657-66.	Excluded by title
48	Mourão J, Neto J, Luís C, Moreno C, Barbosa J, Carvalho J, et al. Dental injury after conventional direct laryngoscopy: a prospective observational study. Anaesthesia. 2013;68(10):1059-65.	Excluded by title
49	Mourão J, Neto J, Viana JS, Carvalho J, Azevedo L, Tavares J. A prospective non-randomised study to compare oral trauma from laryngoscope versus laryngeal mask insertion. Dent Traumatol. 2011;27(2):127-30.	Excluded by title
50	Noma N, Kakigawa H, Kozono Y, Yokota M. Cementum crack formation by repeated loading in vitro. Journal of Periodontology. 2007;78(4):764-9.	Excluded by title
51	Patel KM, Desai RG, Trivedi K, Neuburger PJ, Krishnan S, Potestio CP. Complications of Transesophageal Echocardiography: A Review of Injuries, Risk Factors, and Management. J Cardiothorac Vasc Anesth. 2022;36(8 Pt B):3292-302.	Excluded by title
52	Peebles PJ, Jensen EA, Herrick HM, Wildenhain PJ, Rumpel J, Moussa A, et al. Endotracheal Tube Size Adjustments Within Seven Days of Neonatal Intubation. Pediatrics. 2024;153(4).	Excluded by title
53	Peebles PJ, Reiter JG, Wildenhain PJ, Shults J, Herrick HM, Brajkovic I, et al. Procedural Outcomes of Minimally Invasive Surfactant Therapy: An International Matched Cohort Study. Journal of Pediatrics. 2026;293.	Excluded by title
54	Penarrocha M, Lamas J, Penarrocha M, Garcia B. Immediate maxillary lateral incisor implants with nonocclusal loading provisional crowns. Journal of Prosthodontics. 2008;17(1):55-9.	Excluded by title
55	Pileggi R, Dumsha TC, Myslinksi NR. The reliability of electrical pulp test after concussion injury. Endodontics and Dental Traumatology. 1996;12(1):16-9.	Excluded by title
56	Pizzo G, Licata ME, Giuliana G. Traumatic Dental Injuries: Preventive and Public Health Approaches. Handbook of Dental Care: Diagnostic, Preventive and Restorative Services. Health Care Issues Costs and Access2009. p. 147-62.	Excluded by title
57	Reale SC, Bauer ME, Klumpner TT, Aziz MF, Fields KG, Hurwitz R, et al. Frequency and risk factors for difficult intubation in women undergoing general anesthesia for cesarean delivery: A multicenter retrospective cohort analysis. Anesthesiology. 2022;136(5):697-708.	Excluded by title
58	Reichegger E, Yurttas T, Luedi MM, Urman RD. Closed Claims Analysis of Data for Ambulatory Anesthesia. Anesthesiol Clin. 2026;44(2):197-208.	Excluded by title
59	Ritter L, Mischkowski RA, Neugebauer J, Dreiseidler T, Scheer M, Keeve E, et al. The influence of body mass index, age, implants, and dental restorations on image quality of cone beam computed tomography. Oral Surgery, Oral Medicine, Oral Pathology, Oral Radiology and Endodontology. 2009;108(3):e108-e16.	Excluded by title

60	Silva DCd, Freitas PMd, Calvo AFB, Gimenez T, Zanola M, Imparato JCP. Treatment of Riga-Fede disease using laser therapy: clinical case report. RGO (Porto Alegre). 2017;65(1):87-91.	Excluded by title
61	Silva N, Nourian P, Coelho PG, Rekow ED, Thompson VP. Impact Fracture Resistance of Two Titanium-Abutment Systems Versus a Single-Piece Ceramic Implant. Clinical Implant Dentistry and Related Research. 2011;13(2):168-73.	Excluded by title
62	Skinner MF, Newell EA. Localized hypoplasia of the primary canine in bonobos, orangutans, and gibbons. American Journal of Physical Anthropology. 2003;120(1):61-72.	Excluded by title
63	Sugimoto A, Gonda T, Maeda Y, Ikebe K. Longitudinal Deterioration of Custom-Made Mouthguard Fit During 12 Months of Use in Athletes: Implications for Traumatic Dental Injury Prevention. Dental Traumatology. 2026.	Excluded by title
64	Tang L, Huang J, Guo J, Zhang M, Chen W, Zhao X, et al. Efficacy and Risk Assessment of Lateral Position Endotracheal Intubation Combined with Airway Surface Anesthesia in Gastrointestinal Endoscopic Surgery: a Randomized Controlled Non-Inferiority Study. Therapeutics and clinical risk management. 2025;21:1473-83.	Excluded by title
65	Travessa AM, Dias P, Rosmaninho-Salgado J, Aza-Carmona M, Moldovan O, Díaz-González F, et al. Characterization of three adults and an adolescent with Osteogenesis Imperfecta type VI and a novel founder SERPINF1 variant. European Journal of Medical Genetics. 2023;66(11).	Excluded by title
66	Wang L, Li H, Zhong Y, Ye S, Deng J, Pan T, et al. Comparative Analysis of Hemodynamic Responses and Oropharyngeal Complications in Tracheal Intubation: Evaluating Conventional, Video, and Rigid Video Laryngoscopes Under General Anesthesia. Medical Science Monitor. 2024;30.	Excluded by title
67	Warren M, Kamania J, Dennis AT. Immediate birth: An analysis of women undergoing time critical birth in a tertiary referral obstetric hospital. International Journal of Obstetric Anesthesia. 2016;26:S26.	Excluded by title
68	Warren MH, Kamania J, Dennis AT. Immediate birth – an analysis of women and their babies undergoing time critical birth in a tertiary referral obstetric hospital. International Journal of Obstetric Anesthesia. 2018;33:46-52.	Excluded by title
69	Whyte MP, Tau C, McAlister WH, Zhang X, Novack DV, Preliasco V, et al. Juvenile Paget's disease with heterozygous duplication within TNFRSF11A encoding RANK. Bone. 2014;68:153-61.	Excluded by title
70	Yahyazadehfar M, Arola D. The role of organic proteins on the crack growth resistance of human enamel. Acta Biomaterialia. 2015;19:33-45.	Excluded by title
71	Zhu M, Jia L, Wang Y, Zhang Y, Lv S. Comparative evaluation of adverse drug reactions for tenofovir alafenamide and tenofovir disoproxil fumarate using the FAERS Database: a disproportionality analysis. Expert Opinion on Drug Safety. 2025;24(9):1073-82.	Excluded by title
72	Calado RV, Mendonça Neto PT, Araujo LDC, Pugliesi DMC, Nascimento PBL, Romão DA, et al. Breakdown in Hypomineralization in Deciduous Teeth: An Association between Anthropometric, Orthodontic and Dental Caries Data. Pesquisa Brasileira em Odontopediatria e Clinica Integrada. 2025;25.	Excluded by abstract
73	Cheng R, Lei L, Zhang R, Cai H, Hu T. [Oral Health Status of Obese Children andIntervention Strategies]. Sichuan Da Xue Xue Bao Yi Xue Ban. 2026;57(1):285-90.	Excluded by abstract
74	da Silva IF, Hartwig AD, Goettems ML, Azevedo MS. Comparative Study of Dental Pain Between Children With and Without a History of Maltreatment. Journal of Oral & Facial Pain and Headache. 2019;33(3):287-93.	Excluded by abstract
75	Kao JH. Highlights. Journal of the Formosan Medical Association. 2023;122(12):1231-3.	Excluded by abstract
76	Ludovichetti FS, Signoriello AG, Zuccon A, Padovani S, Mazzoleni S. The Role of Information in Dental Traumatology in Patients during Developmental Age: A Cognitive Investigation. European Journal of Dentistry. 2022;16(2):296-301.	Excluded by abstract
77	Majewski M, Kostrzewska P, Ziółkowska S, Kijek N, Malinowski K. Traumatic dental injuries - practical management guide. Pol Merkur Lekarski. 2022;50(297):216-8.	Excluded by abstract
78	Nishanth G, Aravindha Babu N, Rajesh E, Masthan KMK. Child abuse-dentist perspective. Indian Journal of Public Health Research and Development. 2019;10(11):3152-5.	Excluded by abstract
79	Pahel BT, Rowan-Legg A, Quinonez RB. A Developmental Approach to Pediatric Oral Health. Pediatr Clin North Am. 2018;65(5):885-907.	Excluded by abstract
80	Randall C. Pharmaceutical prescribing for children. Part 1. Introduction and general principles of prescribing for children. Prim Dent Care. 2005;12(4):123-6.	Excluded by abstract
81	Rugg-Gunn AJ, Al-Mohammadi SM, Butler TJ. Malnutrition and developmental defects of enamel in 2- to 6-year-old Saudi boys. Caries research. 1998;32(3):181-92.	Excluded by abstract
82	Tsukiboshi M, Durack C. Traumatic Dental Injuries. Cone Beam Computed Tomography in Endodontics2016. p. 101-18.	Excluded by abstract
83	Turska-Szybka A, Piekoszewska-Ziętek P, Gozdowski D, Olczak-Kowalczyk D, Mądry W. A survey of the oral lesions in newborns and infants: A two-year cross-sectional study. New Medicine. 2020;24(2):104-12.	Excluded by abstract
84	Țuțuianu AV, Moca AE, Ștefănescu T, Slăvescu DA, Șipoș LR, Câlniceanu H, et al. Oro-Dental Characteristics Associated with Pediatric Dental Neglect: A Retrospective Study. Children. 2025;12(9).	Excluded by abstract
85	{Prot} Actrn. Overall efficacy and haemodynamic effects following blind oro-tracheal intubation with Intubating Laryngeal Mask Airway (ILMA) vs conventional direct laryngoscopy guided intubation with Macintosh laryngoscope in patients with normal airway undergoing elective surgery. https://trialsearchwhoint/Trial2aspx?TrialID=ACTRN12612000799853. 2012.	Excluded; protocol
86	{Prot} ChiCtr. A randomized trial comparing non-rotational versus rotational video double-lumen tube intubation in the lateral position for thoracic surgery. https://trialsearchwhoint/Trial2aspx?TrialID=ChiCTR2500115494. 2025.	Excluded; protocol
87	{Prot} ChiCtr. Feasibility study of tracheal intubation in lateral decubitus position using video laryngoscope combined with visual double-lumen tracheal catheter in patients undergoing lobectomy. https://trialsearchwhoint/Trial2aspx?TrialID=ChiCTR2200062989. 2022.	Excluded; protocol
88	{Prot} ChiCtr. The influence of catheter preforming on the effectiveness of video laryngoscopy double lumen bronchial intubation. https://trialsearchwhoint/Trial2aspx?TrialID=ChiCTR2400080906. 2024.	Excluded; protocol
89	{Prot} Ctri. A study comparing Intubation Difficulty Scale in two neck protection methods like neck collar and neck stabilisation by a person during breathing tube insertion using video device in patients under anaesthesia. https://trialsearchwhoint/Trial2aspx?TrialID=CTRI/2025/08/093308. 2025.	Excluded; protocol
90	{Prot} Ctri. Comparing two bougies for airway management in Obese Patients Using a Video Laryngoscope. https://trialsearchwhoint/Trial2aspx?TrialID=CTRI/2025/09/094920. 2025.	Excluded; protocol
91	{Prot} Ctri. Comparision of Airtraq TM video laryngoscope with Macintosh laryngoscope for emergency intubation. https://trialsearchwhoint/Trial2aspx?TrialID=CTRI/2024/05/066825. 2024.	Excluded; protocol
92	{Prot} Ctri. Comparison of two laryngeal mask airway devices as a conduit for endotracheal intubation. https://trialsearchwhoint/Trial2aspx?TrialID=CTRI/2024/01/062087. 2024.	Excluded; protocol
93	{Prot} Ctri. Comparison of videolaryngoscope and intubating laryngeal mask airway for putting endotracheal tube in windpipe with stabilised head and neck in patients undergoing neck surgery. https://trialsearchwhoint/Trial2aspx?TrialID=CTRI/2018/07/015108. 2018.	Excluded; protocol
94	{Prot} Ctri. To compare the duration to intubate a patient with a direct Macintosh laryngoscope and C-mac videolaryngoscope using the Intubation box. https://trialsearchwhoint/Trial2aspx?TrialID=CTRI/2022/09/045457. 2022.	Excluded; protocol
95	{Prot} Ctri. To compare two devices for managing airway during anaesthesia in adult patients undergoing laparoscopic gall bladder surgery. https://trialsearchwhoint/Trial2aspx?TrialID=CTRI/2020/04/024856. 2020.	Excluded; protocol
96	{Prot} Irct20110513006465N. Comparison of the clinical performance of laryngeal mask airway without muscle relaxant with conventional endotracheal intubation method, in maintaining the airway of patients candidates for upper extremity nerve plexus surgery. https://trialsearchwhoint/Trial2aspx?TrialID=IRCT20110513006465N4. 2025.	Excluded; protocol
97	{Prot} Nct. Clinical Performance of Laryseal Clear Versus Ambu Aura 40 Laryngeal Mask Airway for Preschool Children. https://clinicaltrialsgov/study/NCT07553247. 2026.	Excluded; protocol
98	{Prot} Nct. Comparing Between Air-Q Intubating Laryngeal Airway and Ambu® AuraGain™. https://clinicaltrialsgov/show/NCT03130413. 2017.	Excluded; protocol
99	{Prot} Nct. Comparison Between Three Supraglottic Airway Devices ProSeal Laryngeal Mask Airway, Air-Q LMA and Ambu AuraGain. https://clinicaltrialsgov/show/NCT04045899. 2019.	Excluded; protocol
100	{Prot} Nct. Comparison of Haemodynamic Effects During Orotracheal Intubation, by Direct or Indirect Laryngoscopy With Airtraq. https://clinicaltrialsgov/show/NCT03743831. 2018.	Excluded; protocol
101	{Prot} Nct. Comparison of Mask Ventilation in Obese Patients by Differents One Hand Techniques. https://clinicaltrialsgov/study/NCT06817083. 2025.	Excluded; protocol
102	{Prot} Nct. Comparison of Storz C-MAC and D-MAC Orotracheal Intubation Systems in Morbidly Obese Patients. https://clinicaltrialsgov/show/NCT01556061. 2012.	Excluded; protocol
103	{Prot} Nct. Non Invasive Ventilation Combined to High Flow Nasal Cannula Oxygen for Preoxygenation of Hypoxemic ICU Patients. https://clinicaltrialsgov/show/NCT02530957. 2015.	Excluded; protocol
104	{Prot} Nct. US-guided Airway Nerve Block vs. Topical Lidocaine in Video Laryngoscopy for Difficult Bariatric Intubation. https://clinicaltrialsgov/ct2/show/NCT07069686. 2025.	Excluded; protocol
105	{Prot} pm RBR. Direct laryngoscopy or videolaryngoscopy intubation in patients without difficult airway predictors. Randomized Trial in Teaching and Training Center. https://trialsearchwhoint/Trial2aspx?TrialID=RBR-92pm68. 2020.	Excluded; protocol
106	Costa LA, Cantanhede LM, Pereira EM, Crivelini MM, Cuoghi OA, Pereira ALP, et al. Validation of a new experimental model of extrusive luxation on maxillary molars of rats: a histological study. Clinical Oral Investigations. 2018;22(5):1985-94.	Excluded; animal study
107	Andersson L. Epidemiology of Traumatic Dental Injuries. Journal of Endodontics. 2013;39(3):S2-S5.	Excluded; review
108	Bastone EB, Freer TJ, McNamara JR. Epidemiology of dental trauma: A review of the literature. Australian Dental Journal. 2000;45(1):2-9.	Excluded; review
109	Corrêa-Faria P, Martins CC, Bönecker M, Paiva SM, Ramos-Jorge ML, Pordeus IA. Absence of an association between socioeconomic indicators and traumatic dental injury: a systematic review and meta-analysis. Dental Traumatology. 2015;31(4):255-66.	Excluded; review
110	Corrêa-Faria P, Petti S. Are overweight/obese children at risk of traumatic dental injuries? A meta-analysis of observational studies. Dent Traumatol. 2015;31(4):274-82.	Excluded; review
111	Devi KP, Tewari N, O'Connell A, Srivastav S, Rajeswary A, Upadhyay AD, et al. Risk factors associated with traumatic dental injuries in individuals with special healthcare needs-A systematic review and meta-analysis. Dental Traumatology. 2024;40(1):91-110.	Excluded; review
112	Elwadia A, Daud A. Investigating head injury and dental trauma related to shopping carts amongst children under the age of 12 years old: A meta-analysis of observational studies. Journal of Emergency Medicine, Trauma and Acute Care. 2022;2022(SUPPL 1).	Excluded; review
113	Glendor U. Aetiology and risk factors related to traumatic dental injuries--a review of the literature. Dent Traumatol. 2009;25(1):19-31.	Excluded; review
114	Lam R. Epidemiology and outcomes of traumatic dental injuries: a review of the literature. Australian Dental Journal. 2016;61:4-20.	Excluded; review
115	Lima TCD, Coste SC, Fernandes M, Barbato-Ferreira DA, Colosimo EA, Del Fabbro M, et al. Prevalence of traumatic dental injuries in emergency dental services: A systematic review and meta-analysis. Community Dentistry and Oral Epidemiology. 2023;51(2):247-55.	Excluded; review
116	Magno MB, Nadelman P, Leite KLF, Ferreira DM, Pithon MM, Maia LC. Associations and risk factors for dental trauma: A systematic review of systematic reviews. Community Dent Oral Epidemiol. 2020;48(6):447-63.	Excluded; review
117	Magno MB, Neves AB, Ferreira DM, Pithon MM, Maia LC. The relationship of previous dental trauma with new cases of dental trauma. A systematic review and meta-analysis. Dental Traumatology. 2019;35(1):3-14.	Excluded; review
118	Mathus-Vliegen EMH, Nikkel D, Brand HS. Oral aspects of obesity. International Dental Journal. 2007;57(4):249-56.	Excluded; review
119	Mejàre IA, Klingberg G, Mowafi FK, Stecksén-Blicks C, Twetman SHA, Tranæus SH. A systematic map of systematic reviews in pediatric dentistry - What do we really know? PLoS ONE. 2015;10(2).	Excluded; review

120	Patnana AK, Chugh A, Chugh VK, Kumar P, Vanga NRV, Singh S. The prevalence of traumatic dental injuries in primary teeth: A systematic review and meta-analysis. Dental Traumatology. 2021;37(3):383-99.	Excluded; review
121	Petti S. Traumatic dental injuries: epidemiological and therapeutic considerations. A narrative review. Dental Cadmos. 2022;90(8):598-613.	Excluded; review
122	Sabuncuoglu O. Understanding the relationships between breastfeeding, malocclusion, ADHD, sleep-disordered breathing and traumatic dental injuries. Med Hypotheses. 2013;80(3):315-20.	Excluded; review
123	Sharma V, Coleman S, Nixon J, Sharples L, Hamilton-Shield J, Rutter H, et al. A systematic review and meta-analysis estimating the population prevalence of comorbidities in children and adolescents aged 5 to 18 years. Obesity Reviews. 2019;20(10):1341-9.	Excluded; review
124	Soares TRC, Magno MB, Jural LA, Loureiro JM, Chianca TK, de Andrade Risso P, et al. Risk factors for traumatic dental injuries in the Brazilian population: A critical review. Dent Traumatol. 2018;34(6):445-54.	Excluded; review
125	Da Fonseca MA, Hoge C. Extraction of "Killer" teeth: The case of two somali siblings. Journal of Dentistry for Children. 2007;74(2):143-6.	Excluded; case report / series
126	Finucane D, Fleming P, Smith O. Dentoalveolar trauma in a patient with chronic idiopathic thrombocytopenic purpura: A case report. Pediatric Dentistry. 2004;26(4):352-4.	Excluded; case report / series
127	Porter MD, Smith K, Jackson R, Price M, Khoury ZH. Oral and maxillofacial manifestations of Noonan and Costello overlap syndrome: A case report and literature review. Pediatric Dental Journal. 2025;35(3).	Excluded; case report / series
128	Quist M, Chopp D, Wilson CM, Radesky J. Ineffective Homeschooling in a Child with a Learning Disability. J Dev Behav Pediatr. 2019;40(2):152-3.	Excluded; case report / series
129	Ronrang L, Lyngdoh A. Management Modalities of Mandible Fracture and Dental Trauma in Pediatric Patients During Mixed Dentition Phase: A Series of Case Study. Case Reports in Dentistry. 2025;2025(1 C7 - 6662463).	Excluded; case report / series
130	Tewari N, Mathur VP. Smartphones: The new risk factor for traumatic dental and facial injuries in children. Dent Traumatol. 2020;36(1):76-8.	Excluded; case report / series
131	Yildirim Oz G, Ataoğlu H, Kir N, Karaman AI. An alternative method for splinting of traumatized teeth: case reports. Dent Traumatol. 2006;22(6):345-9.	Excluded; case report / series
132	Ahmed I, Kipps C, Fine P. Investigating the effect of mouth guard use on aerobic performance in amateur boxers. Clin Exp Dent Res. 2021;7(5):888-93.	Excluded; no children sample
133	Chatrchaiwiwatana S, Hongsawat K, Siritapetawee M, Ratanasiri A. Dental and Jaw Injuries among Muay Thai Kickboxing Athletes. Journal of the Medical Association of Thailand = Chotmaihet thangphaet. 2016;99:S120-S6.	Excluded; no children sample
134	Coulthard P, Yong S, Adamson L, Warburton A, Worthington HV, Esposito M. Domestic violence screening and intervention programmes for adults with dental or facial injury. Cochrane Database Syst Rev. 2004(2):Cd004486.	Excluded; no children sample
135	Howard J. Dental injuries in adults. British Dental Journal. 2023;234(3):167-.	Excluded; no children sample
136	Leles JLR, dos Santos EJ, Jorge FD, da Silva ET, Leles CR. Risk factors for maxillofacial injuries in a Brazilian emergency hospital sample. Journal of Applied Oral Science. 2010;18(1):23-9.	Excluded; no children sample
137	Liang L, Chuang SK. Trends in Soccer-Related Craniomaxillofacial Injuries, United States 2003-2022. J Oral Maxillofac Surg. 2023;81(12):1495-503.	Excluded; no children sample
138	Minty M, Canceill T, Lê S, Dubois P, Amestoy O, Loubieres P, et al. Oral health and microbiota status in professional rugby players: A case-control study. Journal of Dentistry. 2018;79:53-60.	Excluded; no children sample
139	Noor MFM, Rahman MSHA, Aziz NAA, Tin JTS, Nabil S, Kiem TJ, et al. Oral health assessment of epilepsy patients from a tertiary hospital in Asia. Medical Journal of Malaysia. 2024;79(4):443-51.	Excluded; no children sample
140	Özkal Eminoğlu D, Kaşali K, Gençoğlu C, Ulupinar S, Özbay S, Şeran B, et al. Mouthguard use, hygiene, and maintenance practices among combat and team sports athletes: A comparative study. PLoS ONE. 2025;20(1 January).	Excluded; no children sample
141	Paredes-Corbera ME, Dulanto-Vargas JA, Carranza-Samanez KM. Risks and Consequences of Orofacial Injuries due to Traffic Police-Related Work in Peru. Journal of International Dental and Medical Research. 2025;18(2):691-8.	Excluded; no children sample
142	Perheentupa U, Laukkanen P, Veijola J, Joukamaa M, Järvelin MR, Laitinen J, et al. Increased lifetime prevalence of dental trauma is associated with previous non-dental injuries, mental distress and high alcohol consumption. Dent Traumatol. 2001;17(1):10-6.	Excluded; no children sample
143	Scott N, Thomson WM, Cathro PR. Traumatic dental injuries among New Zealanders: Findings from a national oral health survey. Dent Traumatol. 2020;36(1):25-32.	Excluded; no children sample
144	Abd RK, Alburgaiba MH, Raman V, Rouis S, Mahdi B. Assessment of Some Risk Factors for Dental Caries in a Sample of Children in Nasiriyah City-Iraq. Latin American Journal of Pharmacy. 2023;42(Special Issue):12-7.	Excluded; no TDIs
145	Alfiya M, Shenoy RP, Pasha MI, Junaid, Amanna S. Association Between Body Mass Index and Oral Health Status Among Children: A Cross-Sectional Study Contributing to Policy-Driven Improvements in Children's Oral Health. International Journal of Dentistry. 2026;2026(1).	Excluded; no TDIs
146	Baccetti T, Antonini A. Dentofacial characteristics associated with trauma to maxillary incisors in the mixed dentition. J Clin Pediatr Dent. 1998;22(4):281-4.	Excluded; no TDIs
147	Jürgensen N, Petersen PE. Oral health and the impact of socio-behavioural factors in a cross sectional survey of 12-year old school children in Laos. BMC Oral Health. 2009;9:29.	Excluded; no TDIs
148	Lalloo R, Lucchesi LR, Bisignano C, Castle CD, Dingels ZV, Fox JT, et al. Epidemiology of facial fractures: incidence, prevalence and years lived with disability estimates from the Global Burden of Disease 2017 study. Inj Prev. 2020;26(Supp 1):i27-i35.	Excluded; no TDIs
149	Nicolau B, Marcenes W, Bartley M, Sheiham A. A life course approach to assessing causes of dental caries experience: the relationship between biological, behavioural, socio-economic and psychological conditions and caries in adolescents. Caries Res. 2003;37(5):319-26.	Excluded; no TDIs
150	Troiani V, Ratti E, Gonnella D, Panzeri MC, Palestini P, Cazzaniga E. Dietary and Oral Hygiene Behaviors Associated with Prevalent Caries Status in School-Aged Children of Northern Italy. Nutrients. 2026;18(9).	Excluded; no TDIs
151	Amadori F, Bardellini E, Copeta A, Conti G, Villa V, Majorana A. Dental trauma and bicycle safety: a report in Italian children and adolescents. Acta Odontol Scand. 2017;75(3):227-31.	Excluded; weight not assessed
152	Anirudh BVM, Mathew MG. Prevalence of traumatic dental injuries among children. International Journal of Pharmaceutical Research. 2020;12(2):2343-8.	Excluded; weight not assessed
153	Atabek D, Alaçam A, Aydintug I, Konakoglu G. A retrospective study of traumatic dental injuries. Dental Traumatology. 2014;30(2):154-61.	Excluded; weight not assessed
154	Bendo CB, Paiva SM, Oliveira AC, Goursand D, Torres CS, Pordeus IA, et al. Prevalence and associated factors of traumatic dental injuries in Brazilian schoolchildren. Journal of Public Health Dentistry. 2010;70(4):313-8.	Excluded; weight not assessed
155	Caldas AD, Jr., Burgos MEA. A retrospective study of traumatic dental injuries in a Brazilian dental trauma clinic. Dental Traumatology. 2001;17(6):250-3.	Excluded; weight not assessed
156	da Fonseca RCL, Antunes JLF, Cascaes AM, Bomfim RA. Analysis of the combined risk of oral problems in the oral health-related quality of life of Brazilian adolescents: multilevel approach. Clinical Oral Investigations. 2020;24(2):857-66.	Excluded; weight not assessed
157	da Fonseca RCL, Antunes JLF, Cascaes AM, Bomfim RA. Individual and contextual factors associated with traumatic dental injuries in a population of Brazilian adolescents. Dental Traumatology. 2019;35(3):171-80.	Excluded; weight not assessed
158	Francisco SS, de Souza FJ, Pinheiro É, Murrer RD, Soares AD. Prevalence of Traumatic Dental Injuries and Associated Factors Among Brazilian Schoolchildren. Oral Health & Preventive Dentistry. 2013;11(1):31-8.	Excluded; weight not assessed
159	Glendor U. Epidemiology of traumatic dental injuries - a 12 year review of the literature. Dental Traumatology. 2008;24(6):603-11.	Excluded; weight not assessed
160	Keasberry J, Munyombwe T, Duggal M, Day PF. Summary of: A study of factors that influence the number of visits following traumatic dental injuries. British Dental Journal. 2013;214(11):570-1.	Excluded; weight not assessed
161	Lauridsen E, Hermann NV, Gerds TA, Kreiborg S, Andreasen JO. Pattern of traumatic dental injuries in the permanent dentition among children, adolescents, and adults. Dental Traumatology. 2012;28(5):358-63.	Excluded; weight not assessed
162	López MDR, Cademartori MG, Goettems ML, Demarco FF, Hallal PC, da Costa VPP. Influence of Physical Activity on the Occurrence of Traumatic Dental Injuries in Primary Dentition: A Cohort Study in Southern Brazil. Dental Traumatology. 2025;41(4):419-26.	Excluded; weight not assessed
163	Mahmoodi B, Rahimi-Nedjat R, Weusmann J, Azaripour A, Walter C, Willershausen B. Traumatic dental injuries in a university hospital: a four-year retrospective study. Bmc Oral Health. 2015;15.	Excluded; weight not assessed
164	Michel-Crosato E, Raggio DP, Coloma-Valverde ANJ, Lopez EF, Alvarez-Velasco PL, Medina MV, et al. Oral health of 12-year-old children in Quito, Ecuador: a population-based epidemiological survey. BMC Oral Health. 2019;19(1):184.	Excluded; weight not assessed
165	Özgür B, Ünverdi GE, Güngör HC, McTigue DJ, Casamassimo PS. A 3-Year retrospective study of traumatic dental Injuries to the primary dentition. Dental Traumatology. 2021;37(3):488-96.	Excluded; weight not assessed
166	Percival T, Aylett SE, Pool F, Bloch-Zupan A, Roberts GJ, Lucas VS. Oral health of children with intractable epilepsy attending the UK National Centre for Young People with Epilepsy. Eur Arch Paediatr Dent. 2009;10(1):19-24.	Excluded; weight not assessed
167	Ramos-Jorge ML, Peres MA, Traebert J, Ghisi CZ, de Paiva SM, Pordeus IA, et al. Incidence of dental trauma among adolescents: a prospective cohort study. Dental Traumatology. 2008;24(2):159-63.	Excluded; weight not assessed
168	Sousa SJL, Rezende L, Ibiapina IMP, Ribeiro APD, Bellini M, De Mello DBP, et al. Epidemiological Profile of Dental Trauma: A 13-Year Retrospective Study. International Journal of Dentistry. 2025;2025(1).	Excluded; weight not assessed
169	Årtun J, Al-Azemi R. Social and behavioral risk factors for maxillary incisor trauma in an adolescent Arab population. Dental Traumatology. 2009;25(6):589-93.	Excluded; weight - TDI relation not assessed
170	Sousa de AM, Silva TCL, de Vaigel BC, Pinho RCM, Cimões R. Family functioning and dental trauma, malocclusion and anthropometry in adolescents. Brazilian Journal of Oral Sciences. 2021;20:1-9.	Excluded; weight - TDI relation not assessed
171	Todero SRB, Cavalcante-Leão BL, Fraiz FC, Rebellato NLB, Ferreira FM. The association of childhood sleep problems with the prevalence of traumatic dental injury in schoolchildren. Dent Traumatol. 2019;35(1):41-7.	Excluded; weight - TDI relation not assessed
172	Caldas AF, Soriano EP, Caldas KU, Oliveira MRM. Overjet, lip coverage, and obesity as risk factors related to traumatic dental injuries in Brazilian schoolchildren. Journal of Dental Research. 2003;82:B134-B.	Excluded; no full-text

173	Da Rosa P, Nicolau B, Lambert MA, O'Loughlin J, Tremblay A, Rousseau MC. Exploring socioeconomic gradients in traumatic dental injury among children in Quebec. American Journal of Epidemiology. 2011;173:S146.	Excluded; no full-text
174	Nicolau BF, Marcenes W, Sheoham A. The relationship between obesity and traumatic dental injuries. Journal of Dental Research. 2000;79(5):1199-.	Excluded; no full-text
175	Al-Bajjali TT, Rajab LD. Traumatic dental injuries among 12-year-old Jordanian schoolchildren: an investigation on obesity and other risk factors. BMC Oral Health. 2014;14:101.	Included
176	Alshayeb L, Al-Shiekh MN, Dashash M. Prevalence and Risk Factors of Traumatic Dental Injuries among Conflict-Affected Schoolchildren in Syria (2023-2024). J Epidemiol Glob Health. 2025;15(1):73.	Included
177	Arheiam AA, Elareibi I, Elatrash A, Baker SR. Prevalence and factors associated with traumatic dental injuries among schoolchildren in war-torn Libya. Dent Traumatol. 2020;36(2):185-91.	Included
178	Aswathikutty A, Marcenes W, Stansfeld SA, Bernabé E. Obesity, physical activity and traumatic dental injuries in adolescents from East London. Dent Traumatol. 2017;33(2):137-42.	Included
179	Basha S, Mohamed RN, Al-Thomali Y, Ashour AA, Zahrani FSA, Almutair NE. Traumatic dental injuries in special health care needs children and association with obesity. Ann Saudi Med. 2021;41(1):51-8.	Included
180	Basha S, Mohammad RN, Swamy HS, Sexena V. Association between Traumatic Dental Injury, Obesity, and Socioeconomic Status in 6- and 13-Year-Old Schoolchildren. Soc Work Public Health. 2015;30(4):336-44.	Included
181	Basha S, Mohammad RN, Swamy HS. Incidence of dental trauma among obese adolescents--a 3-year-prospective study. Dent Traumatol. 2015;31(2):125-9.	Included
182	Borges TS, Chaffee BW, Kramer PF, Feldens EG, Vítolo MR, Feldens CA. Relationship between overweight/obesity in the first year of age and traumatic dental injuries in early childhood: Findings from a birth cohort study. Dent Traumatol. 2017;33(6):465-71.	Included
183	Damé-Teixeira N, Alves LS, Susin C, Maltz M. Traumatic dental injury among 12-year-old South Brazilian schoolchildren: prevalence, severity, and risk indicators. Dent Traumatol. 2013;29(1):52-8.	Included
184	Dharmani CKK, Pathak A, Sidhu HS. Prevalence of Traumatic Dental Injuries to Anterior Teeth in 8–12-year-old Schoolchildren of Patiala City, Punjab, India: An Epidemiological Study. International Journal of Clinical Pediatric Dentistry. 2019;12(1):25-9.	Included
185	Goettems ML, Torriani DD, Hallal PC, Correa MB, Demarco FF. Dental trauma: prevalence and risk factors in schoolchildren. Community Dent Oral Epidemiol. 2014;42(6):581-90.	Included
186	Granville-Garcia AF, de Menezes VA, de Lira PI. Dental trauma and associated factors in Brazilian preschoolers. Dent Traumatol. 2006;22(6):318-22.	Included
187	Martins VM, Sousa RV, Rocha ES, Leite RB, Gomes MC, Granville-Garcia AF. Assessment of the association between overweight/obesity and traumatic dental injury among Brazilian schoolchildren. Acta Odontol Latinoam. 2014;27(1):26-32.	Included
188	Martins VM, Sousa RV, Rocha ES, Leite RB, Paiva SM, Granville-Garcia AF. Dental trauma among Brazilian schoolchildren: prevalence, treatment and associated factors. Eur Arch Paediatr Dent. 2012;13(5):232-7.	Included
189	Motta-Rego T, Soares MEC, Souto-Souza D, Souza EA, Paiva SM, Ramos-Jorge ML, et al. Association of the prevalence and severity of untreated traumatic dental injuries with body mass index among Brazilian preschool children. Dent Traumatol. 2022;38(3):206-12.	Included
190	Nicolau B, Marcenes W, Sheiham A. Prevalence, causes and correlates of traumatic dental injuries among 13-year-olds in Brazil. Dent Traumatol. 2001;17(5):213-7.	Included
191	Nicolau B, Marcenes W, Sheiham A. The relationship between traumatic dental injuries and adolescents' development along the life course. Community Dent Oral Epidemiol. 2003;31(4):306-13.	Included
192	Nyquist JM, Phillips C, Stein M, Koroluk LD. Executive function as a risk factor for incisor trauma. Dental Traumatology. 2018;34(4):229-36.	Included
193	Paiva PCP, Paiva HNd, Oliveira Filho PM, Lamounier JA, Zarzar PM. Estudo exploratório sobre a prevalência de traumatismo dentário e obesidade em escolares de 12 anos de idade em Diamantina, Minas Gerais. Arq odontol. 2014;50(01):35-41.	Included
194	Paul B, Acharya S. Measurement of association between malocclusion, nutritional status, and dental trauma in adolescents: A cross-sectional study. Medical Journal Armed Forces India. 2022;78:S232-S7.	Included
195	Petti S, Cairella G, Tarsitani G. Childhood obesity: a risk factor for traumatic injuries to anterior teeth. Endod Dent Traumatol. 1997;13(6):285-8.	Included
196	Primo-Miranda EF, Ramos-Jorge ML, de Souza Morais RL, Silva PCA, Souto-Souza D, Marques LS. Psychomotor Development and Traumatic Dental Injuries in Preschool. Dent Traumatol. 2025;41(6):634-40.	Included
197	Saha R, Guha P, Chandra B, Lahiri PK, Kar S, Basak T, et al. Influence of Childhood Sleeping Disorders on Traumatic Dental Injuries and Body Mass Index among School Children: A Cross-sectional Study. Journal of Clinical and Diagnostic Research. 2026;20(4):ZC54-ZC9.	Included
198	Soares MEC, Primo-Miranda EF, Galo R, Mourão LS, Ramos-Jorge J, Ramos-Jorge ML. Association between obesity and traumatic dental injuries in pre-school children-A case-control study. Dent Traumatol. 2022;38(2):123-8.	Included
199	Soriano EP, Caldas Ade F, Jr., De Carvalho MV, Caldas KU. Relationship between traumatic dental injuries and obesity in Brazilian schoolchildren. Dent Traumatol. 2009;25(5):506-9.	Included
200	Soriano EP, Caldas Ade F, Jr., Diniz De Carvalho MV, Amorim Filho Hde A. Prevalence and risk factors related to traumatic dental injuries in Brazilian schoolchildren. Dent Traumatol. 2007;23(4):232-40.	Included
201	Soriano EP, Caldas AF, Jr., Góes PS. Risk factors related to traumatic dental injuries in Brazilian schoolchildren. Dent Traumatol. 2004;20(5):246-50.	Included
202	Tapias MA, Jiménez-García R, Lamas F, Gil AA. Prevalence of traumatic crown fractures to permanent incisors in a childhood population: Móstoles, Spain. Dent Traumatol. 2003;19(3):119-22.	Included
203	Teixeira ND. Epidemiologia do traumatismo dentário em escolares de 12 anos em Porto Alegre-RS Brasil. 2011. p. 85-.	Included
204	Tsuchiya S, Tsuchiya M, Momma H, Sekiguchi T, Kuroki K, Kanazawa K, et al. Factors associated with sports-related dental injuries among young athletes: a cross-sectional study in Miyagi prefecture. BMC Oral Health. 2017;17(1):168.	Included
205	Vijaykumar S, Guna Shekhar M, Vijayakumar R. Traumatic dental injuries and its relation to overweight among Indian school children living in an urban area. Journal of Clinical and Diagnostic Research. 2013;7(11):2631-3.	Included

TDI, traumatic dental injury.

Table S3. Detailed risk of bias assessment of included studies with the JBI tool.

	Q1	Q2	Q3	Q4	Q5	Q6	Q7	Q8	Summary
	**Were the same criteria used for identification of normoweight - overweight/obese patients?**	**Was overweight/obesity measured in a standard, valid and reliable way?**	**Was overweight/obesity measured in the same way for patients with/without TDI?**	**Were confounding factors identified?**	**Were TDIs assessed in a standard, valid and reliable way?**	**Was the exposure period of interest long enough?**	**Was the sample size adequate (>350)?**	**Was appropriate statistical analysis used, including confounder adjustment?**	**Overall risk of bias**
Al-Bajjali 2014	**Yes**	**Yes**	**Yes**	**Yes**	**Yes**	**Yes**	**Yes**	**No**	**High**
Alshayeb 2025	**Yes**	**Yes**	**Yes**	**Partly**	**Yes**	**Yes**	**Yes**	**No**	**High**
Arheiam 2020	**Yes**	**Yes**	**Yes**	**Yes**	**Yes**	**Yes**	**Yes**	**Yes**	**Low**
Aswathikutty 2017	**Yes**	**Yes**	**Yes**	**Yes**	**Yes**	**Yes**	**Yes**	**Yes**	**Low**
Basha 2015a SWPH	**Yes**	**Yes**	**Yes**	**Yes**	**Yes**	**Yes**	**Yes**	**Yes**	**Low**
Basha 2015b DT	**Yes**	**Yes**	**Yes**	**Yes**	**Yes**	**Yes**	**Yes**	**Yes**	**Low**
Basha 2021	**Yes**	**Yes**	**Yes**	**Yes**	**Yes**	**Yes**	**Yes**	**Yes**	**Low**
Borges 2017	**Yes**	**Yes**	**Yes**	**No**	**Yes**	**No**	**Yes**	**Yes**	**High**
Dharmani 2019	**Yes**	**Yes**	**Yes**	**Yes**	**Yes**	**Yes**	**Yes**	**No**	**High**
Goettems 2014	**Yes**	**Yes**	**Yes**	**Yes**	**Yes**	**Yes**	**Yes**	**Yes**	**Low**
Granville-Garcia 2006	**Yes**	**Yes**	**Yes**	**No**	**Yes**	**No**	**Yes**	**Partly**	**High**
Martins 2012; 2014	**Yes**	**Yes**	**Yes**	**Yes**	**Yes**	**Yes**	**Yes**	**Partly**	**Moderate**
Motta-Rego 2022	**Yes**	**Yes**	**Yes**	**Partly**	**Yes**	**No**	**No**	**Partly**	**High**
Nicolau 2001; 2003	**Yes**	**Yes**	**Yes**	**No**	**Yes**	**Yes**	**Yes**	**Partly**	**High**
Nyquist 2018	**No**	**Yes**	**Yes**	**Yes**	**No**	**Yes**	**No**	**No**	**High**
Paiva 2014	**Yes**	**Yes**	**Yes**	**Yes**	**Yes**	**Yes**	**No**	**No**	**High**
Paul 2022	**Yes**	**Yes**	**Yes**	**No**	**Yes**	**Yes**	**Yes**	**Partly**	**High**
Petti 1997	**Yes**	**Yes**	**Yes**	**Yes**	**Yes**	**Yes**	**Yes**	**Partly**	
Primo-Miranda 2025	**Yes**	**Yes**	**Yes**	**Yes**	**Yes**	**No**	**No**	**Partly**	**High**
Saha 2026	**Yes**	**Yes**	**Yes**	**No**	**Yes**	**Yes**	**Yes**	**No**	**High**
Soares 2022	**No**	**Yes**	**Yes**	**Partly**	**Yes**	**No**	**Yes**	**Partly**	**High**
Soriano 2004	**Yes**	**No**	**Yes**	**Partly**	**Yes**	**Yes**	**No**		**High**
Soriano 2007; 2009	**Yes**	**No**	**Yes**	**No**	**Yes**	**Yes**	**Yes**	**Partly**	**High**
Tapias 2003	**Yes**	**Yes**	**Yes**	**Partly**	**No**	**Yes**	**Yes**	**Partly**	**High**
Teixeira 2011; Dame-Teixeira 2013	**Yes**	**Yes**	**Yes**	**Yes**	**Yes**	**Yes**	**Yes**	**No**	**High**
Tsuchiya 2017	**Yes**	**Yes**	**Yes**	**Partly**	**No**	**Yes**	**Yes**	**Partly**	**High**
Vijaykumar 2013	**Yes**	**Yes**	**Yes**	**No**	**No**	**Yes**	**Yes**	**Partly**	**High**

TDI, traumatic dental injury.

Table S4. Details of included studies in each meta-analysis.

Analysis	Nr	Study	Data	Control	Experimental	Comparison
Overweigh/obesity	2	Alshayeb 2025	raw	NW	OWOB	NW vs OWOB
	4	Aswathikutty 2017	adj	NW	OWOB	NW vs OWOB
	5	Basha 2015a	raw	NW	OWOB	NW vs OWOB
	6	Basha 2015b	adj	NW	OWOB	NW vs OWOB
	7	Basha 2021	raw	NW	OWOB	NW vs OWOB
	8	Borges 2017	adj	NW	OWOB	NW vs OWOB
	18	Primo-Miranda 2025	adj	NW	OWOB	NW vs OWOB
	19	Saha 2026	raw	NW	OWOB	NW vs OWOB
	24	Teixeira 2011	raw	NW	OWOB	NW vs OWOB
	25	Tsuchiya 2017	raw	NW	OWOB	NW vs OWOB
	14	Nicolau 2001	raw	UWNW	OW	UWNW vs OW
	3	Arheiam 2020	adj	UWNW	OWOB	UWNW vs OWOB
	10	Goettems 2014	adj	UWNW	OWOB	UWNW vs OWOB
	11	Granville-Garcia 2006	adj	UWNW	OWOB	UWNW vs OWOB
	12	Martins 2012	raw	UWNW	OWOB	UWNW vs OWOB
	13	Motta-Rego 2022	adj	UWNW	OWOB	UWNW vs OWOB
	15	Paiva 2014	raw	UWNW	OWOB	UWNW vs OWOB
	16	Paul 2022	adj	UWNW	OWOB	UWNW vs OWOB
	20	Soares 2022	adj	UWNW	OWOB	UWNW vs OWOB
	23	Tapias 2003	adj	UWNW	OWOB	UWNW vs OWOB
	26	Vijaykumar 2013	raw	UWNW	OWOB	UWNW vs OWOB
	1	Al-Bajjali 2014	raw	UWNWOW	OB	UWNWOW vs OB
	9	Dharmani 2019	raw	UWNWOW	OB	UWNWOW vs OB
	17	Petti 1997	adj	UWNWOW	OB	UWNWOW vs OB
	21	Soriano 2004	raw	UWNWOW	OB	UWNWOW vs OB
	22	Soriano 2007	raw	UWNWOW	OB	UWNWOW vs OB
Overweight	1	Alshayeb 2025	raw	NW	OW	NW vs OW
	2	Aswathikutty 2017	adj	NW	OW	NW vs OW
	3	Basha 2015a	raw	NW	OW	NW vs OW
	4	Basha 2015b	raw	NW	OW	NW vs OW
	5	Basha 2021	raw	NW	OW	NW vs OW
	6	Primo-Miranda 2025	adj	NW	OW	NW vs OW
	7	Saha 2026	raw	NW	OW	NW vs OW
	9	Teixeira 2011	raw	NW	OW	NW vs OW
	8	Soares 2022	adj	UWNW	OW	UWNW vs OW
	10	Nicolau 2001	raw	UWNW	OW	UWNW vs OW
Obesity	1	Alshayeb 2025	raw	NW	OB	NW vs OB
	2	Aswathikutty 2017	adj	NW	OB	NW vs OB
	3	Basha 2015a	raw	NW	OB	NW vs OB
	4	Basha 2015b	raw	NW	OB	NW vs OB
	5	Basha 2021	raw	NW	OB	NW vs OB
	6	Primo-Miranda 2025	adj	NW	OB	NW vs OB
	7	Saha 2026	raw	NW	OB	NW vs OB
	9	Teixeira 2011	raw	NW	OB	NW vs OB
	8	Soares 2022	adj	UWNW	OB	UWNW vs OB

adj, adjusted for confounders; NW, normoweight; OB, obese; OW, overweight; UW, underweight; vs, versus.

Table S5. Subgroup analysis according to the dentitions of included studies.

	**Deciduous dentition**		**Mixed dentition**		**Permanent dentition**		**Various dentitions**		
**Comparison**	**Studies (patients)**	**OR (95% CI)**	**Studies (patients)**	**OR (95% CI)**	**Studies (patients)**	**OR (95% CI)**	**Studies (patients)**	**OR (95% CI)**	**$P_{subsets*}$**
Overweight / obese	5 (4101)	1.95 (1.34, 2.85)	1 (770)	1.11 (0.76, 1.62)	12 (8886)	1.63 (1.12, 2.39)	8 (10015)	1.17 (0.89, 1.54)	0.42
Overweight	2 (621)	1.28 (0.71, 2.33)	-	-	5 (3674)	1.05 (0.44, 2.50)	3 (2248)	1.22 (0.50, 2.98)	0.52
Obese	2 (578)	1.76 (0.46, 6.68)	-	-	4 (2836)	1.15 (0.53, 2.49)	3 (2051)	1.72 (1.40, 2.11)	0.11

CI, confidence interval; OR, odds ratio.

* pertaining only to the comparison between permanent versus deciduous dentition, since the other subsets were very inconsistent.

Table S6. Sensitivity analysis according to the sample size of included studies.

	Sample <500 patients			500 patients ≤ Sample <1000 patients			Sample ≥ 1000 patients		
**Comparison**	**Studies (patients)**	**OR (95% CI)**		**Studies (patients)**	**OR (95% CI)**		**Studies (patients)**	**OR (95% CI)**	$P_{subsets}$
Overweight / obese	7 (1963)	1.48 (0.95, 2.30)		10 (6990)	1.75 (1.28, 2.41)		9 (14819)	1.24 (0.84, 1.84)	0.29
Overweight	4 (1317)	1.12 (0.68, 1.86)		4 (2582)	1.43 (0.46, 4.39)		2 (2644)	0.91 (0.84, 0.99)	0.19
Obese	3 (842)	1.74 (1.31, 2.31)		4 (2277)	1.46 (0.77, 2.75)		2 (2346)	1.13 (0.02, 77.11)	0.33

CI, confidence interval; OR, odds ratio.

Table S7. Sensitivity analysis according to the design of included studies.

	Cross-sectional studies			Cohort studies			Case-control studies		
**Comparison**	**Studies (patients)**	**OR (95% CI)**		**Studies (patients)**	**OR (95% CI)**		**Studies (patients)**	**OR (95% CI)**	$P_{subsets}$
Overweight / obese	22 (21518)	1.48 (1.18, 1.84)		3 (1748)	1.56 (0.40, 6.05)		1 (506)	1.52 (1.03, 2.25)	0.98
Overweight	7 (4968)	1.35 (0.94, 1.92)		2 (1130)	0.53 (0.36, 0.77)		1 (445)	1.19 (0.70, 2.00)	<0.001
Obese	6 (3994)	1.53 (0.95, 2.46)		2 (1039)	1.22 (0.82, 1.80)		1 (432)	2.05 (1.15, 3.68)	0.11

CI, confidence interval; OR, odds ratio.

Table S8. Sensitivity analysis according to the traumatic dental injury criteria of included studies.

	**Overweight / obese**			**Overweight / obese**			**Overweight / obese**	
**Criteria**	**Studies (patients)**	**OR (95% CI)**		**Studies (patients)**	**OR (95% CI)**		**Studies (patients)**	**OR (95% CI)**
Andreasen	14 (8902)	1.53 (1.21, 1.94)		6 (3536)	1.06 (0.68, 1.65)		6 (3150)	1.65 (1.39, 1.95)
Glendor	1 (598)	0.94 (0.62, 1.43)		1 (463)	0.56 (0.26, 1.19)		1 (518)	1.18 (0.72, 1.93)
IADT	1 (600)	2.78 (1.60, 4.81)		1 (592)	2.50 (1.39, 4.50)		1 (549)	5.49 (1.29, 23.29)
UKCDHS	5 (6530)	1.56 (0.92, 2.63)		2 (1952)	1.27 (0.01, >100)		1 (1248)	0.83 (0.57, 1.22)
Other	5 (7142)	1.26 (0.53, 2.96)		-	-		-	-
**$P_{subsets}$**		0.04			0.01			0.001

CI, confidence interval; IADT, International Association of Dental Traumatology; OR, odds ratio; UKCDHS, United Kingdom Children's Dental Health Survey.

Figure S1. Contour-enhanced funnel plot for the effect of overweight / obesity on traumatic dental injuries in children.

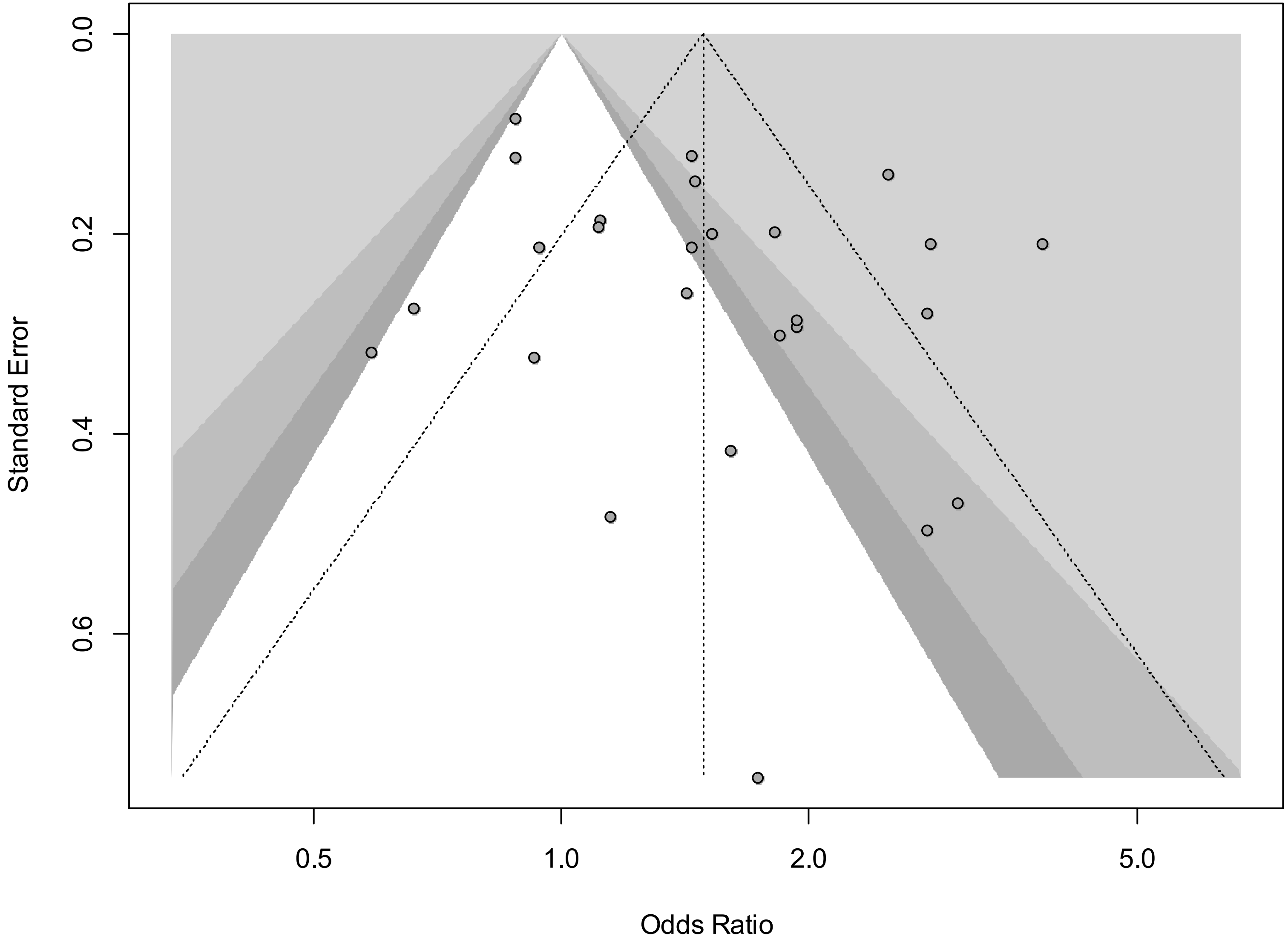

Figure S2. Contour-enhanced funnel plot for the effect of overweight on traumatic dental injuries in children.

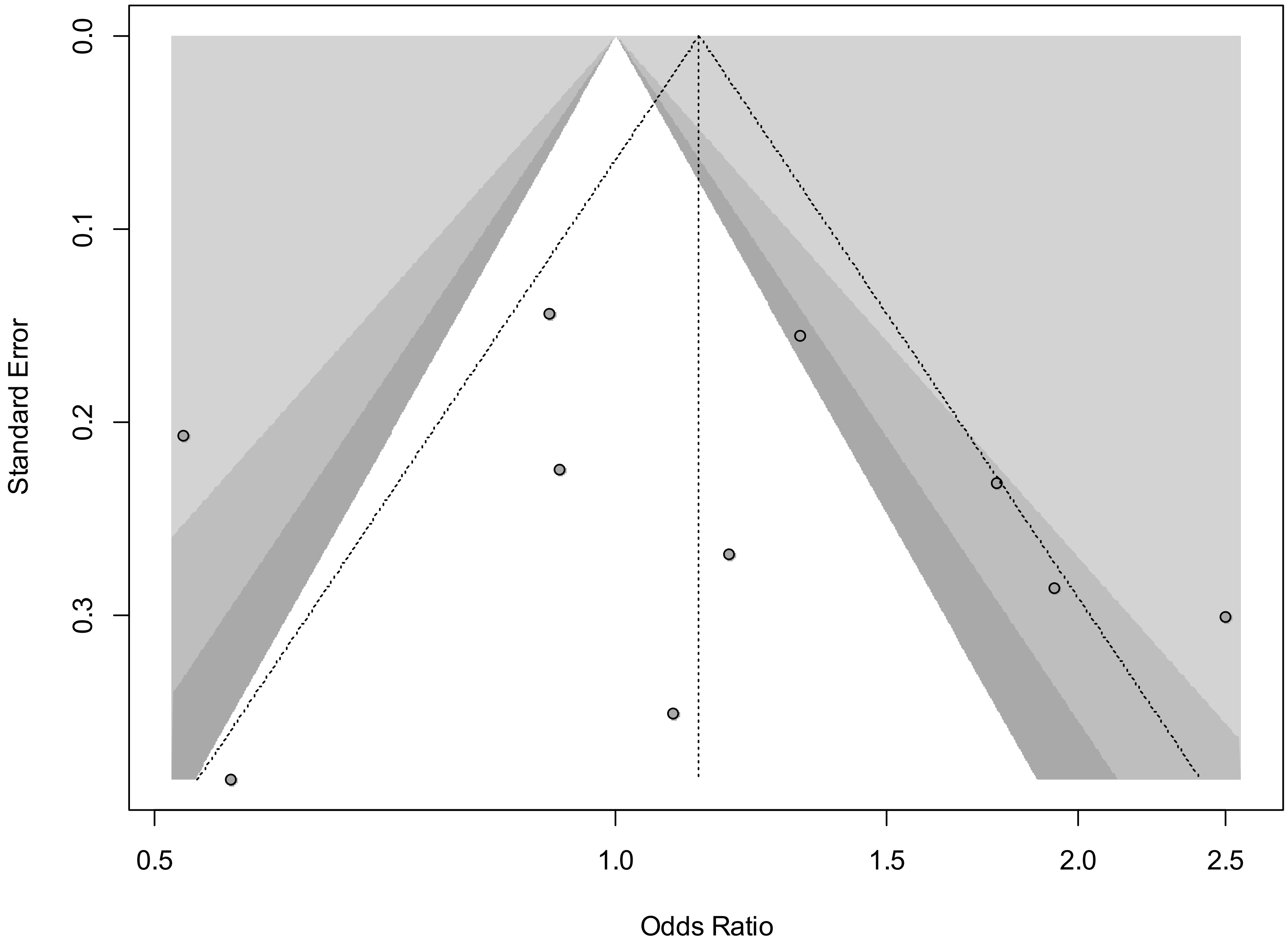

Supplement: Supplementary file 1 [file jcm-15-07749-s001.zip › jcm-4537629-supplementary.pdf]
